# Happy but Unequal: Differences in Subjective Well-Being across Individuals and Space in Colombia

**DOI:** 10.1007/s11482-021-09954-2

**Published:** 2021-07-27

**Authors:** Martijn Burger, Martijn Hendriks, Elena Ianchovichina

**Affiliations:** 1 Department of Applied Economics, Erasmus University, Rotterdam, Tinbergen Institute and Academic Director at the Erasmus Happiness Economics Research Organisation (EHERO), P.O. Box 1738, 3000 DR Rotterdam, The Netherlands; 2 Department of Applied Economics, Erasmus University, Rotterdam and a Senior Researcher at the Erasmus Happiness Economics Research Organisation (EHERO), Room M5-39, Van der Goot Building, Burg. Oudlaan 50, 3000 PA Rotterdam, The Netherlands; 3 Office of the Chief Economist, Latin America and the Caribbean Region of the World Bank, 1818 H Street NW, Washington, DC USA

**Keywords:** Subjective well-being, Life satisfaction, Happiness, Perceived welfare, Income inequality, Inequality in subjective well-being, Spatial inequality, Colombia, D60, D63, I31, Z13

## Abstract

Despite being on average a relatively happy country, Colombia has a high level of inequality in subjective well-being (SWB). Using Gallup World Poll data for the period from 2010 to 2018, this paper tests the direction and strength of association of a range of objective and subjective factors with SWB and explains differences in SWB across individuals and space. The perceived welfare of the average Colombian is mainly influenced by conditions and expectations related to economic opportunities and education. However, quantile regressions, reveal substantial differences in the domains that matter to those at the bottom and top of the experienced welfare distribution. Standard-of-living improvements, housing affordability, and civic engagement matter more to the most fortunate top 20%, while having education, a job, sufficient income, economic security, and digital connectivity are much more strongly associated with the well-being of the bottom 20%. The life domains that matter more to the unhappiest respondents also explain the majority of the spatial differences in perceived welfare between residents in urban and rural areas as well as core and peripheral regions. Policy actions aimed at closing the gaps in these areas have the potential to increase well-being and reduce inequality in Colombia.

## Introduction

Since 1971, when the king of Bhutan proclaimed that ‘Gross National Happiness’ is more important than ‘Gross Domestic Product’, the idea that the Gross Domestic Product (GDP) of a country is an insufficient measure to accurately track quality-of-life changes within a country has gained international support. In 2012 the General Assembly of the United Nations adopted a resolution that governments should try to increase the happiness of their citizens. In the years leading to the resolution many governments had started paying explicit attention to subjective well-being (SWB) measures that go beyond the GDP and other standard objective measures to track changes in the quality of life (Stiglitz et al., 2009). Some governments have taken this idea further. Since 2015 the United Kingdom’s *What Works Centre for Wellbeing* has been collecting and disseminating information on ways to promote SWB-based policy (Frijters et al., 2020). In 2019, New Zealand’s Prime Minister Jacinda Ardern expressed her preference for gauging the long-term impact of policies on people’s happiness rather than relying on short-term indicators (Dalziel, 2019). Similar initiatives have started in Iceland, Scotland, Finland, and Wales.^1^ In parallel with practice, empirical evidence has helped to mainstream and broaden the use of SWB data by policy makers (Graham et al., 2018). Studies have shown that standard objective measures traditionally used to assess changes in poverty, inequality and welfare cannot adequately capture changes in quality of life (Deaton, 2008; Graham & Lora, 2010; Arampatzi et al. 2018) and emerging social discontent (Witte et al., 2020). These types of issues are captured by SWB data (Veenhoven, 2012), which are also increasingly perceived as a consistent and meaningful way of measuring perceived or experienced welfare (Senik, 2011).^2^

**Fig. 1:**
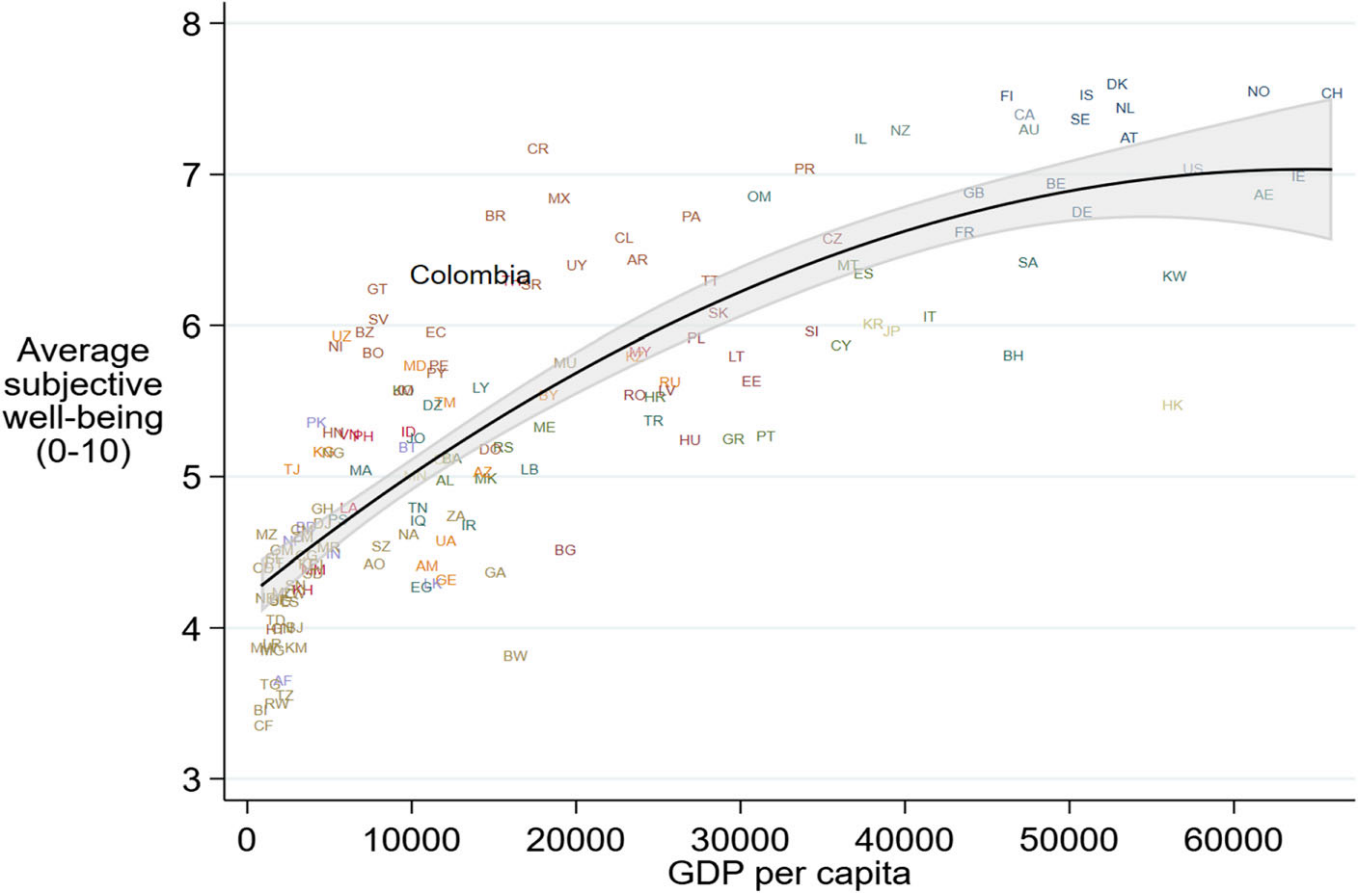
SWB by economic development at the country level. Notes: *N* = 153 countries. Sampling weights and two-letter country codes are used. No control variables. Log GDP per capita at Purchasing Power Parities (PPP) using constant 2017 international $ is derived from the World Development Indicators. For both indicators the average of the 2010–2018 period is taken. Non-linear regression line and 95% confidence interval shown. SWB taken from the Gallup World Poll and based on the question: *Please imagine a ladder, with steps numbered from 0 at the bottom to 10 at the top. The top of the ladder represents the best possible life for you and the bottom of the ladder represents the worst possible life for you. On which step of the ladder would you say you personally feel you stand at this time?* For Colombia, the average SWB is 6.34 and the GDP per capita is $13,594

**Fig. 2:**
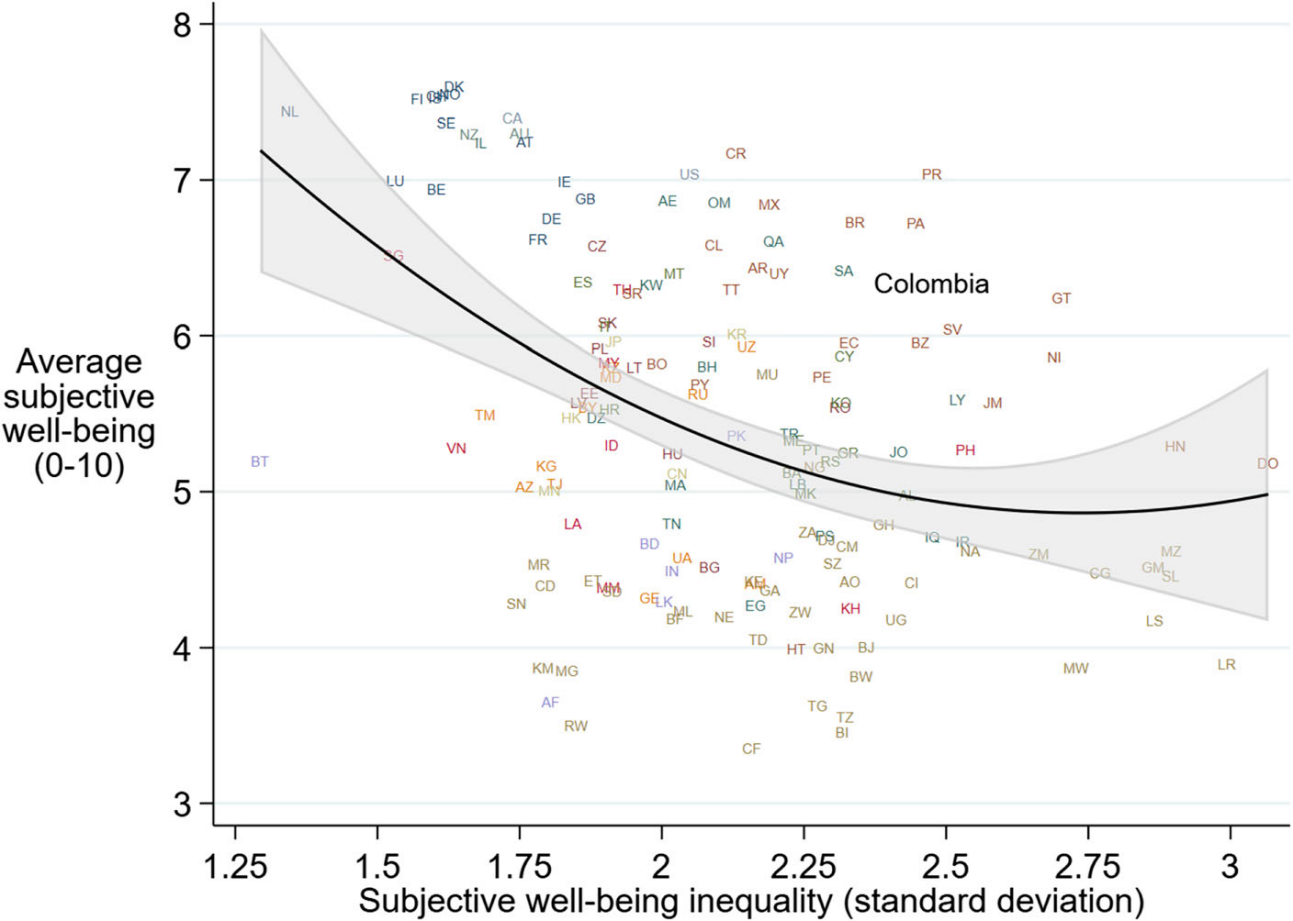
SWB by SWB inequality at the country level in the world. Source: Gallup World Poll (2010–2018). Notes: *N* = 156 countries. Sampling weights and two-letter country codes are used. No control variables. Subjective well-being inequality is measured using the method proposed by Veenhoven and Kalmijn (2005). Non-linear regression line and 95% confidence interval shown

**Fig. 3:**
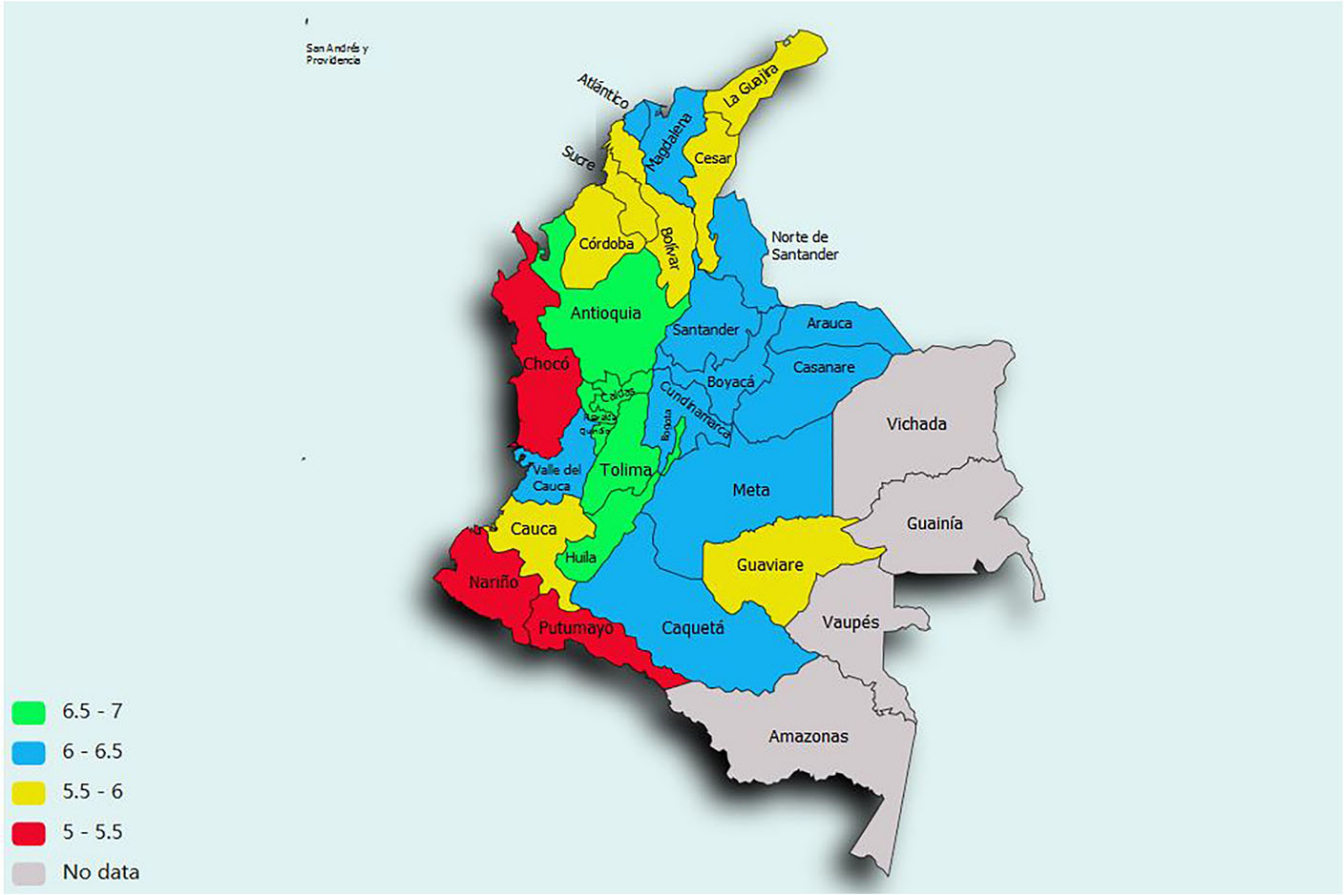
SWB in Colombia across regions. Source: Gallup World Poll (2010–2018). Notes: Sampling weights are used. No control variables

**Fig. 4:**
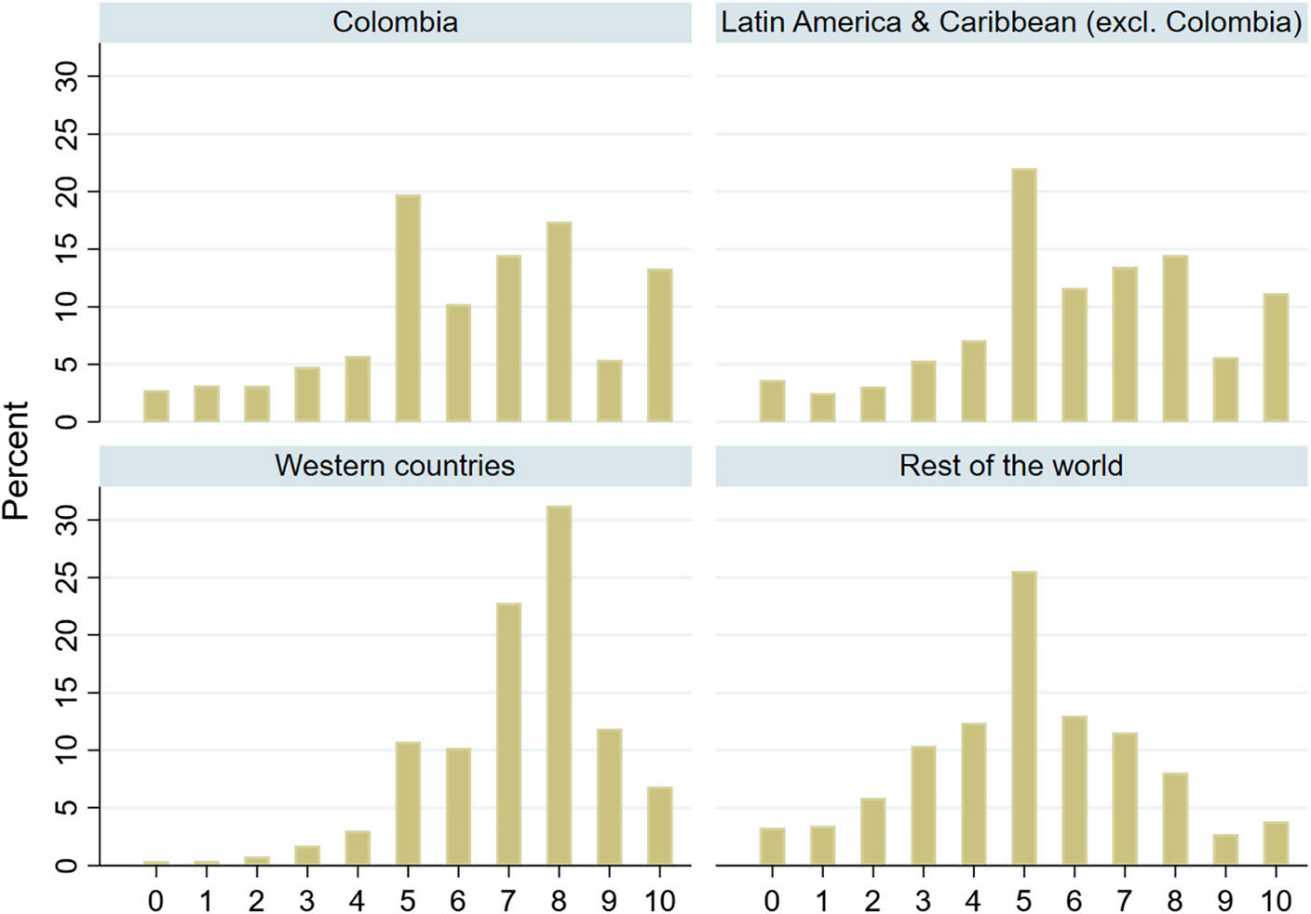
SWB distribution *Note:* Western countries includes Northern & Western Europe, Australia, New Zealand, United States, and Canada

**Fig. 5:**
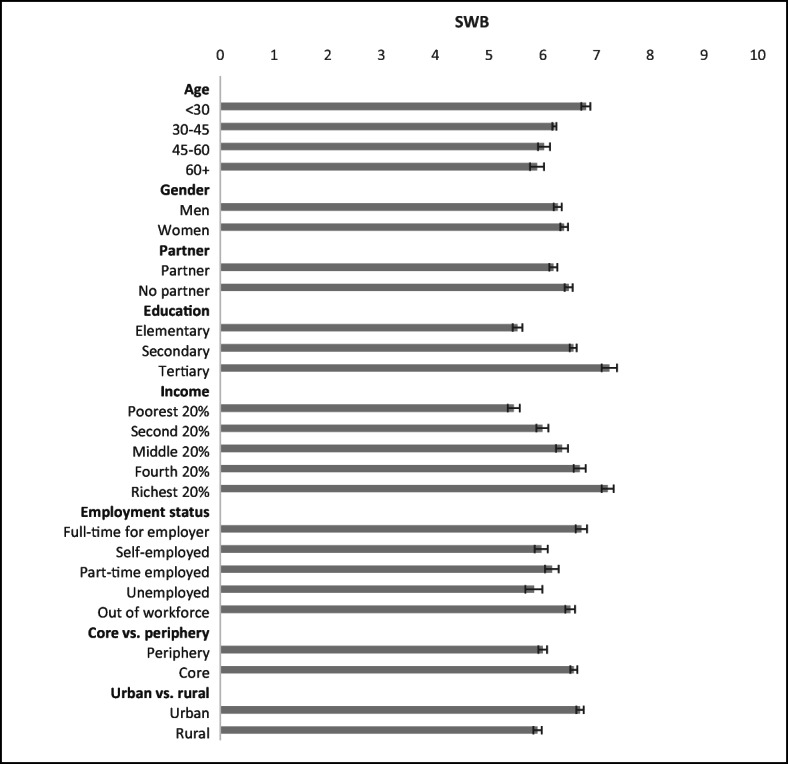
SWB by demographic group. *Notes:* 95% confidence intervals shown. *N* = 8917

**Fig. 6:**
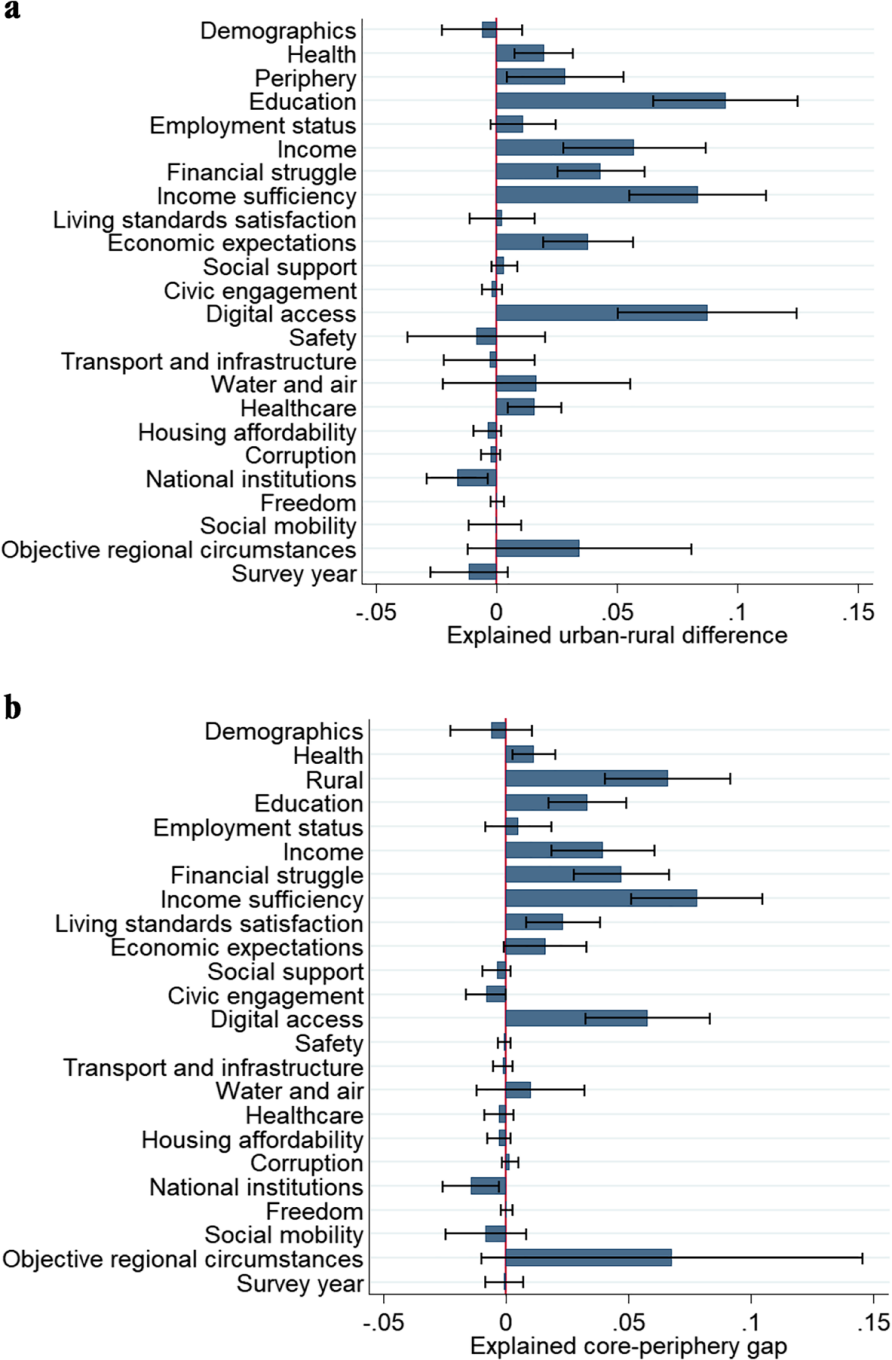
**a** Factors explaining the urban-rural difference in SWB **b** Factors explaining the core-periphery difference in SWB. Note: Explained urban-rural difference is 55% and explained core-periphery difference is 73%. For graphical representation some variables have been grouped. Demographics: age, gender, maritial status, children, religion, and migrant status; Health: health problems and pain; Economic expectations: personal economic optimism and optimism about economic climate; Transport and infrastructure: satisfaction with public transport and satisfaction with roads and highways; Social mobility: Social mobility is possible and satisfied with poverty policy; Objective regional circumstances: Regional GDP per capita, regional institutional quality, and share of Venezuelan migrants

**Fig. 7:**
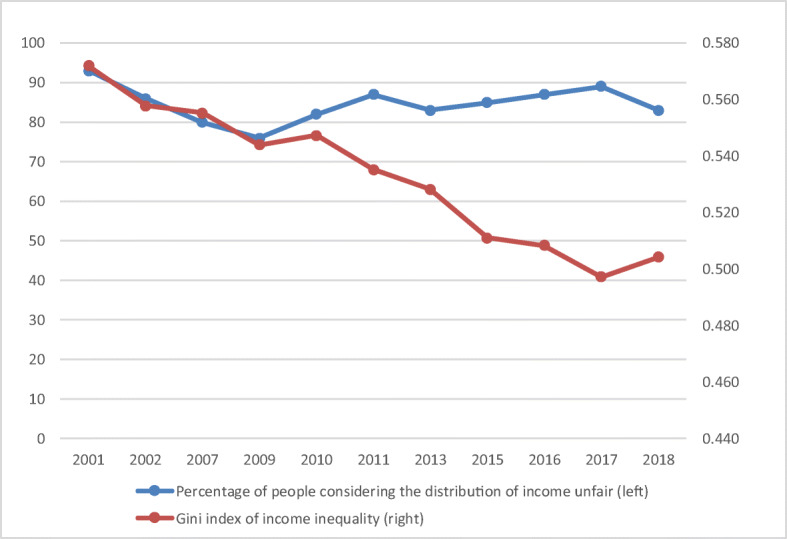
Income inequality and perceptions about income inequality. (Source: Latinobarómetro and World Bank)

**Fig. 8:**
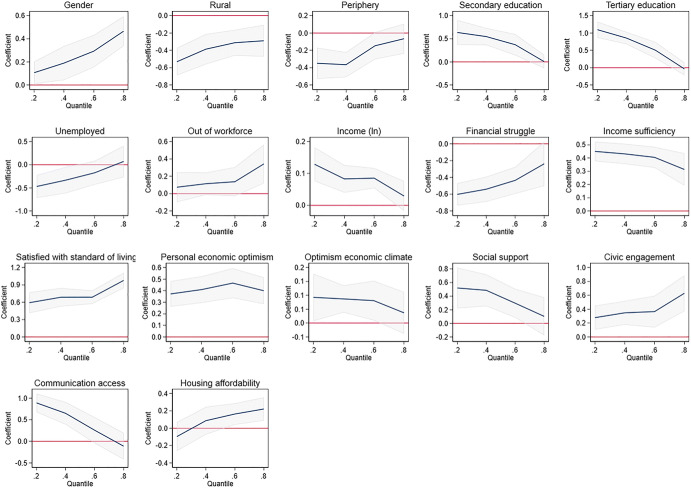
Graphs related to quantile regressions in Table 4

**Table 1:** Sample descriptives of the GWP data for Colombia, 2010–2018

Variable	N	Mean	SD	Min	Max
SWB	8917	6.34	2.48	0	10
Age	8911	39.23	17.43	15	100
Female	8917	0.52		0	1
Has a partner	8902	0.50		0	1
Has children under 15	8908	0.57		0	1
Immigrant	8916	0.01		0	1
Religious	8852	0.86		0	1
Has health problems	8914	0.20		0	1
Had physical pain yesterday	8912	0.32		0	1
Lives in the periphery	8917	0.41		0	1
Lives in a rural area	8886	0.45		0	1
Elementary education	8896	0.30		0	1
Secondary education	8896	0.58		0	1
Tertiary education	8896	0.12		0	1
Full-time employed for employer	8917	0.24		0	1
Self-employed	8917	0.18		0	1
Part-time employed	8917	0.16		0	1
Unemployed	8917	0.10		0	1
Out of workforce	8917	0.32		0	1

**Table 2:** Geographical representativeness analysis sample

Region	% of population in sample (average 2010–2018)	% of total population (average 2010–2018)	Difference between columns 1 and 2
Bogotá	16.7	16.3	0.4
Antioquia	13.1	13.4	−0.3
Valle del Cauca	10.5	9.6	0.9
Cundinamarca	4.6	5.5	−0.9
Atlántico	5.9	5.1	0.8
Santander	5.4	4.3	1.1
Bolívar	4.8	4.4	0.4
Córdoba	3.2	3.5	−0.3
Nariño	4.1	3.6	0.5
Norte de Santander	3.0	2.8	−0.2
Cauca	3.2	2.9	0.3
Magdalena	2.7	2.6	0.1
Tolima	2.7	2.9	−0.2
Boyacá	2.5	2.7	0.2
Cesar	2.4	2.1	−0.3
Huila	2.4	2.4	0
Meta	2.1	2.0	0.1
Caldas	2.0	2.1	−0.1
Risaralda	2.0	2.0	0
La Guajira	1.4	2.0	−0.6
Sucre	1.3	1.8	−0.5
Caquetá	1.3	1.0	0.3
Quindío	0.9	1.2	−0.3
Chocó	0.9	1.0	−0.1
Casanare	0.4	0.7	−0.3
Putumayo	0.4	0.7	−0.3
Arauca	0.2	0.5	−0.3
Vichada	0.1	0.1	0
Vaupés	0.1	0.1	0
Guaviare	0	0.2	−0.2
Amazonas	0	0.2	−0.2
San Andrés y Providencia	0	0.2	−0.2
Guainía	0	0.1	−0.1

Source: DANE (National Administrative Department of Statistics) and Gallup World Poll

**Table 3:** Regression analyses with life satisfaction model

	(1) Personal characteristics		(2) Subjective economic situation		(3) Social capital and digital access		(4) Community basics and safety		(5) Perceptions of public policy and institutions		(6) Objective regional characteristics		(7) Full model	
Dependent variable: SWB	Coef.	Tstat	Coef.	Tstat	Coef.	Tstat	Coef.	Tstat	Coef.	Tstat	Coef.	Tstat	Coef.	Tstat
Age	−0.07**	−8.3	−0.03**	−3.6	−0.06**	−6.9	−0.06**	−7.6	−0.06**	−7.2	−0.07**	−8.6	−0.02**	−2.9
Age^2^	0.07**	6.7	0.03**	3.4	0.06**	5.8	0.06**	5.8	0.06**	5.9	0.07**	7.0	0.02*	2.5
Female	0.28**	4.9	0.27**	4.5	0.27**	4.8	0.29**	4.7	0.30**	5.6	0.28**	4.8	0.27**	4.6
Has a partner	0.06	0.6	−0.01	−0.1	0.06	0.7	0.06	0.8	0.04	0.5	0.05	0.6	0.00	0.0
Has children under 15	−0.05	−0.7	−0.02	−0.3	−0.05	−0.7	−0.05	−0.7	−0.06	−0.9	−0.04	−0.6	−0.03	−0.4
Religious	0.24*	2.5	0.15	1.5	0.21*	2.2	0.12	1.3	0.10	1.1	0.23*	2.4	0.07	0.8
Immigrant	−0.67	−1.2	−0.73	−1.4	−0.71	−1.3	−0.86	−1.6	−0.79	−1.5	−0.67	−1.2	−0.89	−1.7
Has health problems	−0.54**	−5.7	−0.28**	−3.0	−0.51**	−5.5	−0.49**	−5.3	−0.52**	−5.2	−0.54**	−5.7	−0.27**	−3.0
Had physical pain yesterday	−0.37**	−6.0	−0.10	−1.5	−0.33**	−5.2	−0.29**	−4.3	−0.30**	−4.5	−0.37**	−5.9	−0.07	−1.0
Lives in a rural area	−0.47**	−5.6	−0.45**	−5.9	−0.41**	−4.9	−0.49**	−5.1	−0.50**	−5.7	−0.47**	−5.8	−0.39**	−4.7
Lives in the periphery	−0.30*	−2.7	−0.21*	−2.2	−0.29*	−2.8	−0.31**	−2.8	−0.36**	−3.1	−0.25*	−2.1	−0.17	−1.8
Education level														
*Elementary*	Ref.	Ref.	Ref.	Ref.	Ref.	Ref.	Ref.	Ref.	Ref.	Ref.	Ref.	Ref.	Ref.	Ref.
*Secondary*	0.57**	8.1	0.46**	6.2	0.44**	5.8	0.66**	9.9	0.66**	10.2	0.56**	8.3	0.44**	5.8
*Tertiary*	1.03**	9.0	0.74**	7.1	0.74**	5.7	1.20**	10.9	1.16**	11.2	1.01**	8.9	0.67**	5.9
Employment status														
*Full-time employed for employer*	Ref.	Ref.	Ref.	Ref.	Ref.	Ref.	Ref.	Ref.	Ref.	Ref.	Ref.	Ref.	Ref.	Ref.
*Self-employed*	−0.25**	−4.3	−0.19**	−3.3	−0.25**	−4.7	−0.23**	−3.4	−0.26**	−4.4	−0.24**	−4.2	−0.20**	−3.7
*Part-time employed*	−0.14	−1.7	0.04	0.5	−0.14	−1.8	−0.15	−2.0	−0.14	−1.8	−0.12	−1.4	0.03	0.4
*Unemployed*	−0.62**	−3.8	−0.16	−1.1	−0.57**	−3.7	−0.56**	−3.5	−0.58**	−3.7	−0.62**	−3.9	−0.16	−1.1
Out of workforce	0.11	1.7	0.16	2.3	0.12	1.8	0.09	1.2	0.09	1.4	0.11	1.5	0.15*	2.0
Per capita income (ln)	0.21**	5.8	0.09**	3.4	0.18**	5.3	0.20**	5.9	0.21**	5.8	0.21**	6.1	0.08**	3.5
Financial struggle			−0.51**	−7.1									−0.47**	−6.2
Income sufficiency			0.38**	10.2									0.34**	9.5
Satisfied with standard of living			0.69**	7.3									0.58**	6.3
Personal economic optimism			0.45**	8.1									0.41**	7.9
Optimism about economic climate			0.12**	3.2									0.05	1.4
Social support					0.86**	6.7							0.36**	2.8
Civic engagement					0.34**	3.9							0.25*	2.4
Digital access					0.78**	4.4							0.52**	3.2
Safety							0.36**	4.4					0.05	0.5
Satisfied with public transportation							0.03	0.4					−0.00	−0.0
Satisfied with roads and highways							0.16*	2.5					0.16**	2.8
Satisfied with quality of air							0.03	0.4					−0.01	−0.2
Satisfied with quality of water							0.10	0.9					0.04	0.4
Satisfied with health care							0.35**	5.2					0.21**	3.6
Satisfied with housing affordability							0.35**	4.5					0.10	1.3
Corruption									−0.15	−1.4			−0.11	−1.2
Confidence in national institutions									0.60**	5.8			0.30**	3.1
Satisfied with freedom									0.21*	2.5			−0.03	−0.3
Social mobility is possible									0.47**	5.2			0.19	2.0
Satisfied with poverty policy									0.05	0.8			−0.10	−1.4
Regional GDP per capita (ln)											0.12	0.9	0.13	1.4
Regional share of Venezuelan migrants											−0.05*	−2.7	−0.03*	−2.4
Regional institutional quality											−0.00	−0.2	−0.01	−1.0
Year dummies	Yes		Yes		Yes		Yes		Yes		Yes		Yes	
Observations	7080		7080		7080		7080		7080		7080		7080	
R^2^	0.129		0.222		0.150		0.153		0.145		0.133		0.238	

Notes: * *p* < 0.05, ** *p* < 0.01. Regressions are conducted with robust standard errors clustered at the regional level. Sample weights used. ‘Ref.’ stands for reference group and ‘tstat’ for T-statistic

**Table 4:** Lewbel IV estimates

Dependent variable: SWB	WLS Coef.	Lewbel Coef.	Kleibergen-Paap rk Wald F statistic	Hansen J test (*p* value)
Financial struggle	−0.48**	−0.99**	15.86	0.56
Income sufficiency	0.34**	0.48**	33.24	0.89
Satisfied with standard of living	0.58**	0.15	17.03	0.69
Personal economic optimism	0.41**	0.47**	21.70	0.17
Optimism about economic climate	0.05	0.17**	26.91	0.09
Social support	0.36**	0.49*	21.08	0.75
Civic engagement	0.25*	0.20	14.68	0.19
Digital access	0.52**	1.13**	31.62	0.58
Safety	0.05	0.30	25.97	0.70
Satisfied with public transportation	−0.00	−0.23	29.13	0.55
Satisfied with roads and highways	0.15*	0.40	24.31	0.94
Satisfied with quality of air	−0.01	0.36	22.34	0.73
Satisfied with quality of water	0.04	0.28	26.55	0.38
Satisfied with health care	0.21**	0.30	25.98	0.21
Satisfied with housing affordability	0.10	−0.05	24.06	0.32
*Corruption*	−0.11	*0.37*	*10.36*	*0.36*
Confidence in national institutions	0.31**	0.95**	14.77	0.32
Satisfied with freedom	−0.02	−0.08	23.79	0.26
Social mobility is possible	0.19	0.12	28.77	0.34
Satisfied with poverty policy	−0.10	−0.31	24.63	0.62

Notes: * p < 0.05, ** p < 0.01. Regressions are conducted with robust standard errors clustered at the regional level. Sample weights used. Every estimate in the Lewbel column is based a separate Lewbel IV model, in which only the variable that is represented in that row was instrumented. The critical value for the Kleibergen-Paap (10% maximal IV relative bias) for the Stock and Yogo test is 11.10 (Stock & Yogo, 2002). Estimations with potentially weak instruments are highlighted in *italics*

**Table 5:** Relative importance of different variables in explaining differences in SWB

Variable	Beta
Income sufficiency	.123
Personal economic optimism	.120
*Satisfied with standard of living*	*.101*
Tertiary education	.092
Secondary education	.088
Rural	−.078
Financial struggle	−.077
Digital access	.063
Female	.055
Income	.051
Social support	.044
Has health problems	−.043
Confidence in national institutions	.042
*Satisfied with health care*	*.042*
*Satisfied with roads and highways*	*.033*
*Civic engagement*	*.032*
Self-employed	−.031
Out of workforce	.028
Share of Venezuelan migrants	−.026

Note: Ordered by relative strength; table only includes standardized regression coefficients of significant variables in Table 3 (Column 7). The subjective variables indicated in italics are insignificant in the Lewbel IV estimations. As standardized regression coefficients do not work well for interaction terms, the variable age is omitted

**Table 6:** Quantile regressions of reduced full model

	(1) Q20	(2) Q40	(3) Q60	(4) Q80	(5) Equality of coefficients (p value)
Age	−0.00	−0.02*	−0.03**	−0.01	0.15
Age^2^	−0.01	0.03**	0.03**	0.01	0.06
Female	0.11	0.19**	0.29**	0.47**	0.02
Religious	−0.00	0.02	0.09	0.15	0.65
Has health problems	−0.20	−0.26**	−0.37**	−0.31**	0.17
Had physical pain yesterday	−0.14*	−0.04	−0.06	−0.14*	0.24
Lives in a rural area	−0.53**	−0.39**	−0.31**	−0.29**	0.01
Lives in the periphery	−0.35**	−0.37**	−0.15*	−0.07	0.00
Education level					
Elementary	Ref.	Ref.	Ref.	Ref.	
Secondary	0.64**	0.55**	0.37**	0.01	0.00
Tertiary	1.10**	0.85**	0.50**	−0.03	0.00
Employment status					
Full-time employed for employer	Ref.	Ref.	Ref.	Ref.	
Self-employed	−0.31**	−0.23*	−0.17	−0.07	0.49
Part-time employed	−0.07	−0.12	−0.09	0.04	0.25
Unemployed	−0.47**	−0.33*	−0.17	0.07	0.09
Out of workforce	0.07	0.11	0.14	0.34**	0.04
Per capita income (ln)	0.13**	0.08*	0.09*	0.03	0.07
Financial struggle	−0.60**	−0.54**	−0.44**	−0.24	0.01
Income sufficiency	0.45**	0.43**	0.41**	0.31**	0.07
Satisfied with standard of living	0.59**	0.69**	0.69**	0.98**	0.00
Personal economic optimism	0.37**	0.41**	0.47**	0.40**	0.48
Optimism about economic climate	0.09**	0.09**	0.08*	0.04	0.71
Social support	0.52**	0.49**	0.30**	0.10	0.08
Civic engagement	0.28*	0.35**	0.37**	0.63**	0.07
Digital access	0.89**	0.65**	0.26**	−0.11	0.00
Safety	0.16	0.05	−0.01	−0.09	0.34
Satisfied with roads and highways	0.12	0.13	0.24**	0.33**	0.18
Satisfied with health care	0.18*	0.10	0.09	0.12	0.51
Satisfied with housing affordability	−0.10	0.09	0.16*	0.22**	0.00
Confidence in national institutions	0.10	0.10	0.21	0.32*	0.50
Satisfied with freedom	−0.02	0.03	−0.03	0.01	0.76
Social mobility is possible	0.17	0.08	0.12	0.16	0.72
Regional GDP per capita (ln)	0.29**	0.14	0.19	0.13	0.40
Share of Venezuelan migrants	−0.03	−0.04**	−0.06**	−0.03	0.20

*N* = 7080. * p < 0.05, ** p < 0.01. Columns 1–4 report how the explanatory variables relate to SWB at the 20th percentile (col. 1), the 40th percentile (col. 2), the 60th percentile (col. 3), and the 80th percentile (col. 4). Column 5 reports the *P* value from an ANOVA test that the coefficients at the 20th, 40th, 60th, and 80th percentile are equivalent. Dark grey indicates that factor is stronger associated with SWB of unhappiest quintiles, while light grey indicates that factor is stronger associated with SWB of happiest quintiles

**Table 7:** The unhappiest individuals are poorer and worry more about income

SWB quintile	Average household income (International $)	Income sufficiency (1–4)	% Financial struggle	% Satisfied with standard of living
1 (4 or lower)	1964	2.1	52%	55%
2 (5)	3057	2.5	38%	70%
3 (6 or 7)	3948	2.8	25%	77%
4 (8)	5265	3.0	19%	87%
5 (9 or 10)	4180	2.9	25%	89%

Source: Gallup World Poll

**Table 8:** Life comparisons of rural, urban, core, and peripheral areas

Variable	Urban	Rural	Core	Periphery
Age	39	39	38	39
Female	50	53	51	52
% has a partner	49	53	48	54
% has children under 15	55	61	55	61
% immigrants	0.6	0.6	0.8	0.4
% religious	83	90	84	89
% has health problems	17	22	18	21
% had physical pain yesterday	29	33	30	32
% lives in the periphery	34	51	N.A.	N.A.
% lives in a rural area	N.A.	N.A.	37	54
% elementary education	21	38	26	32
% secondary education	62	54	59	58
% tertiary education	17	8	15	11
% full-time employed for employer	28	20	28	20
% self-employed	16	20	17	18
% part-time employed	16	18	16	18
% unemployed	10	11	10	12
% out of workforce	31	31	30	32
Per capita income	4900	2444	4696	2582
% in financial struggle	28	36	27	37
Income sufficiency (1–4)	2.8	2.5	2.8	2.5
% satisfied with standard of living	76	76	78	74
Personal economic optimism (1–3)	2.6	2.5	2.6	2.5
Optimism about economic climate (1–3)	2.1	2.1	2.1	2.1
% having social support	90	90	90	91
Civic engagement index (0–1)	0.35	0.35	0.33	0.37
Digital access index (0–1)	0.78	0.63	0.76	0.66
Safety index (0–1)	0.55	0.69	0.60	0.62
% Satisfied with public transportation	56	70	62	62
% Satisfied with roads and highways	45	48	46	47
% Satisfied with quality of air	58	78	63	72
% Satisfied with quality of water	83	66	80	69
% Satisfied with health care	50	42	46	47
% Satisfied with housing affordability	48	52	49	52
Corruption index (0–1)	0.84	0.82	0.83	0.84
Confidence in national institutions index (0–1)	0.33	0.38	0.33	0.38
% satisfied with freedom	81	83	82	83
% considers social mobility possible	87	90	85	93
% satisfied with poverty policy	29	35	29	36
Regional GDP per capita	14,722	11,325	15,632	9853
% Share of Venezuelan migrants	1.0	0.9	1.0	1.0
Regional institutional quality	73	74	74	73

N = 7080; Highlighted columns denote a statistically significant (p < 0.05) difference

**Table 9:** Variable descriptions and definitions

Variable	Question	Scale
SWB	Please imagine a ladder, with steps numbered from 0 at the bottom to 10 at the top. The top of the ladder represents the best possible life for you and the bottom of the ladder represents the worst possible life for you. On which step of the ladder would you say you personally feel you stand at this time?	0 (worst possible life) – 10 (best possible life)
Personal characteristics: demographics		
Age & age^2^/100		Age in years
Gender		Male/female
Has a partner	Dummy taking the value of 1 if the respondent is married or has a domestic partner, and 0 otherwise	1 = yes; 0 = no
Has children under 15	Dummy taking the value of 1 if the respondent has children under 15 living in one’s household, and 0 otherwise	1 = yes; 0 = no
Immigrant	Were you born in this country, or not?	1 = no; 0 = yes
Religious	Is religion an important part of your daily life?	1 = yes; 0 = no
Personal characteristics: health		
Has health problems	Do you have any health problems that prevent you from doing any of the things people your age normally can do?	1 = yes; 0 = no
Had physical pain yesterday	Did you experience the following feelings during a lot of the day yesterday? How about physical pain?	1 = yes; 0 = no
Personal characteristics: place of residence		
Lives in the periphery	Dummy taking the value of 1 for people residing in North Caribbean, South West, or National Territory, and taking the value of 0 for people residing in Bogotá, Central East, or Antioquia/Eje Cafeter	1 = no; 0 = yes
Lives in a rural area	The respondent’s self-reported type of settlement: (1) Rural area or farm; (2) Small town or village; (3) Large city; (4) Suburb of a large city. ‘Rural’ is defined as individuals in categories (1) and (2) and “urban” as individuals in categories (3) and (4)	1 = rural; 0 = urban
Personal characteristics: education		
Education level	What is your highest completed level of education? Elementary: Completed elementary education or less (up to 8 years of basic education); Secondary: Completed secondary education and up to 3 years tertiary education (nine to 15 years of education); Tertiary: Completed 4 years of education beyond “high school” and/or received a 4-year college degree	1 = elementary 2 = secondary 3 = tertiary
Personal characteristics: objective economic situation		
Employment status	Divided in 5 categories based on a series of questions. (1) employed full-time for an employer; (2) self-employed, (3) part-time employed, (4) unemployed, and (5) out of workforce	
Per capita income	Per capita income (reported household income divided by household size)	International dollars
Subjective economic situation		
Financial struggle	Index with 2 equally weighted items. Have there been times in the past 12 months when you did not have enough money to:	1 = yes; 0 = no
	1. Buy food that you or your family needed?	
	2. Provide adequate shelter or housing for you and your family?	
Income sufficiency	Which one of these phrases comes closest to your own feelings about your household’s income these days?	1–4
	1 = living comfortably on present income	
	2 = getting by on present income	
	3 = finding it difficult on present income	
	4 = finding it very difficult on present income	
Satisfied with standard of living	Are you satisfied or dissatisfied with your standard of living, all the things you can buy and do?	1 = satisfied; 0 = dissatisfied
Economic optimism		
Personal economic optimism	Right now, do you feel your standard of living is getting better or getting worse?	1 = getting worse 2 = the same 3 = getting better
Optimism about economic climate	Right now, do you think that economic conditions in the city or area where you live, as a whole, are getting better or getting worse?	1 = getting worse 2 = the same 3 = getting better
Social capital		
Social support	If you were in trouble, do you have relatives or friends you can count on to help you whenever you need them, or not?	1 = yes; 0 = no
Civic engagement	Index with 3 equally weighted items. Have you done any of the following in the past month? How about:	1 = yes; 0 = no
	1. Donated money to a charity?	
	2. Volunteered your time to an organization?	
	3. Helped a stranger or someone you didn’t know who needed help?	
Digital access		
Digital access	Index with 2 equally weighted items.	1 = yes; 0 = no
	1. Do you have a landline telephone in your home or a mobile phone that you use to make and receive personal calls?	
	2. Do you have access to the internet in any way, whether on a mobile phone, a computer, or some other device?	
Local conditions: air & water		
Satisfied with quality of air	In your city or area where you live, are you satisfied or dissatisfied with:	1 = satisfied; 0 = dissatisfied
Satisfied with quality of water	The quality of air?	
	The quality of water?	
Local conditions: infrastructure		
Satisfied with public transportation	In your city or area where you live, are you satisfied or dissatisfied with:	1 = satisfied; 0 = dissatisfied
	The public transportation systems?	
Satisfied with roads and highways	The roads and highways?	
Satisfied with health care	The availability of quality health care?	
Local conditions: housing affordability		
Satisfied with housing affordability	In your city or area where you live, are you satisfied or dissatisfied with the availability of good affordable housing?	1 = satisfied; 0 = dissatisfied
Local conditions: safety		
Safety	Index with 4 equally weighted items.	1 = yes; 0 = no
	1. Do you feel safe walking alone at night in the city or area where you live?	
	2. In the city or area where you live, do you have confidence in the local police force?	
	3. Within the last 12 months, have you had money or property stolen from you or another household member?	
	4. Within the past 12 months, have you been assaulted or mugged?	
National conditions: corruption		
Corruption	Index with 2 equally weighted items.	1 = yes; 0 = no
	1. Is corruption widespread within businesses located in Colombia, or not?	
	2. Is corruption widespread throughout the government in Colombia, or not?	
National conditions: confidence in national institutions		
Confidence in national institutions	Index with 4 equally weighted items. Do you have confidence in each of the following, or not?	1 = yes; 0 = no
	1. How about the military?	
	2. How about the judicial system and courts?	
	3. How about the national government?	
	4. How about the honesty of elections?	
National conditions: freedom		
Satisfied with freedom	In Colombia are you satisfied or dissatisfied with your freedom to choose what you do with your life?	1 = satisfied; 0 = dissatisfied
National conditions: social mobility and poverty		
Social mobility is possible	Can people in this country get ahead by working hard, or not?	1 = yes; 0 = no
Satisfied with poverty policy	In Colombia, are you satisfied or dissatisfied with efforts to deal with the poor?	1 = satisfied; 0 = dissatisfied
Objective regional circumstances		
Regional GDP per capita (ln)	The natural logarithm of regional GDP per capita at Purchasing Power Parities (PPP) using constant 2017 international dollars derived from the OECD regional statistics database	
Regional institutional quality	The average of the institutional environment score and the institutional performance score (r = 0.93) in the Departmental Institutional Environment and Performance Survey (EDID) collected by DANE*	
Share of Venezuelan migrants	For the period before 2015, the share of Venezuelan migrants in a region is computed based on the regional number of Venezuelan immigrants in 2005; for the period after 2015, the 2015 value is used	

*https://www.dane.gov.co/index.php/en/statistics-by-topic-1/government/institutional-environment-and-performance-at-departmental-level-edid

**Table 10:** Quantile regressions – robustness analyses

	(1) Q20	(2) Q40	(3) Q60	(4) Q80	(5) Equality of coefficients (p value)
**Model 1**					
Age	−0.06**	−0.08**	−0.08**	−0.07**	0.21
Age^2^	0.05**	0.08**	0.08**	0.08**	0.26
Female	0.18	0.21**	0.34**	0.61**	0.00
Has a partner	0.17	0.17*	0.11	0.05	0.65
Has children under 15	0.02	−0.13	−0.07	−0.03	0.10
Religious	0.15	0.08	0.27**	0.37**	0.01
Immigrant	−0.81**	−1.04**	−0.54	−0.24	0.15
Has health problems	−0.51**	−0.57**	−0.61**	−0.56**	0.85
Had physical pain yesterday	−0.37**	−0.30**	−0.38**	−0.45**	0.37
Lives in a rural area	−0.61**	−0.46**	−0.41**	−0.27**	0.00
Lives in the periphery	−0.50**	−0.48**	−0.29**	−0.08	0.00
Education level					
Elementary	Ref.	Ref.	Ref.	Ref.	
Secondary	1.04**	0.72**	0.57**	−0.04	0.00
Tertiary	1.77**	1.44**	1.03**	0.06	0.00
Employment status					
Full-time employed for employer	Ref.	Ref.	Ref.	Ref.	
Self-employed	−0.37*	−0.20*	−0.12	0.04	0.11
Part-time employed	−0.19	−0.25*	−0.29**	−0.08	0.26
Unemployed	−1.04**	−0.83**	−0.65**	−0.30	0.00
Out of workforce	0.05	0.13	0.20*	0.34*	0.15
Per capita income (ln)	0.37**	0.25**	0.19**	0.13**	0.00
**Model 2**					
Financial struggle	−0.68**	−0.58**	−0.52**	−0.19	0.01
Income sufficiency	0.48**	0.46**	0.43**	0.34**	0.16
Satisfied with standard of living	0.70**	0.82**	0.78**	1.03**	0.00
Personal economic optimism	0.37**	0.46**	0.51**	0.49**	0.37
Optimism about economic climate	0.14**	0.12**	0.12**	0.16**	0.68
**Model 3**					
Social support	1.21**	1.01**	0.84**	0.58**	0.00
Civic engagement	0.34**	0.37**	0.45**	0.76**	0.02
Digital access	0.90**	0.95**	0.67**	0.17	0.00
**Model 4**					
Safety	0.54**	0.39**	0.25*	0.31*	0.07
Satisfied with public transportation	0.16	0.08	0.13	0.15	0.42
Satisfied with roads and highways	0.11	0.22**	0.20**	0.29**	0.41
Satisfied with quality of air	−0.00	−0.00	−0.06	0.05	0.42
Satisfied with quality of water	0.09	0.19*	0.13**	0.08	0.47
Satisfied with health care	0.33**	0.24**	0.26**	0.30**	0.57
Satisfied with housing affordability	0.11	0.36**	0.47**	0.51**	0.00
**Model 5**					
Corruption	−0.09	−0.19	−0.30**	−0.28	0.66
Confidence in national institutions	0.31*	0.51**	0.56**	0.64**	0.40
Satisfied with freedom	0.32**	0.43**	0.21*	0.17	0.04
Social mobility is possible	0.56**	0.37**	0.34**	0.40**	0.37
Satisfied with poverty policy	0.10	0.06	0.23*	0.45**	0.01
**Model 6**					
Regional GDP per capita (ln)	0.22	0.18	0.11	0.06	0.70
Share of Venezuelan migrants	−0.08**	−0.07**	−0.07**	−0.08*	0.92
Regional institutional quality	−0.01*	−0.01	0.00	0.01	0.08

Note: Dark grey indicates that the factor is more strongly associated with SWB of the unhappiest quintiles, while light grey indicates that the factor is more strongly associated with SWB of the happiest quintiles

**Table 11:** Extreme bounds analysis

Explanatory variable	Mean coefficient	Mean standard error	CDF(0)	Fraction significant (*p* < .05)	Conclusion
Age	−0.05	0.01	100	100	Robust
Age^2^	0.04	0.01	100	98	Robust
Female	0.27	0.06	100	100	Robust
Has a partner	0.05	0.06	78	4	Fragile
Has children under 15	−0.06	0.06	95	10	Fragile
Religious	0.16	0.08	70	71	Fragile
Immigrant	−0.75	0.41	96	36	Fragile
Has health problems	−0.57	0.08	100	100	Robust
Had physical pain yesterday	−0.40	0.06	100	99	Robust
Lives in a rural area	−0.62	0.06	100	100	Robust
Lives in the periphery	−0.40	0.06	100	99	Robust
Education level					
*Elementary*	Ref.	Ref.	Ref.	Ref.	
*Secondary*	0.71	0.08	100	100	Robust
*Tertiary*	1.33	0.09	100	100	Robust
Employment status					
*Full-time employed for employer*	Ref.	Ref.	Ref.	Ref.	
*Self-employed*	−0.35	0.09	100	100	Robust
*Part-time employed*	−0.32	0.09	71	30	Fragile
*Unemployed*	−0.76	0.12	100	99	Robust
*Out of workforce*	−0.07	0.08	73	19	Fragile
Per capita income (ln)	0.23	0.02	100	100	Robust
Financial struggle	−1.10	0.08	100	100	Robust
Income sufficiency	0.72	0.04	100	100	Robust
Satisfied with standard of living	1.18	0.07	100	100	Robust
Personal economic optimism	0.74	0.05	100	100	Robust
Optimism about economic climate	0.27	0.03	100	99	Robust
Social support	0.79	0.10	100	100	Robust
Civic engagement	0.38	0.10	100	100	Robust
Digital access	1.13	0.11	100	100	Robust
Safety	0.32	0.09	94	78	Fragile
Satisfied with public transportation	0.10	0.06	78	37	Fragile
Satisfied with roads and highways	0.24	0.06	100	100	Robust
Satisfied with quality of air	−0.07	0.06	76	19	Fragile
Satisfied with quality of water	0.36	0.07	100	80	Fragile
Satisfied with health care	0.37	0.06	100	100	Robust
Satisfied with housing affordability	0.33	0.06	100	95	Robust
Corruption	−0.11	0.09	81	18	Fragile
Confidence in national institutions	0.21	0.09	100	85	Fragile
Satisfied with freedom	0.14	0.07	89	52	Fragile
Social mobility is possible	0.31	0.09	98	88	Fragile
Satisfied with poverty policy	0.10	0.07	77	39	Fragile
Regional GDP per capita (ln)	0.33	0.07	100	80	Fragile
Regional share of Venezuelan migrants	−0.04	0.02	98	66	Fragile
Regional institutional quality	−0.01	0.01	81	0	Fragile

Note: Column 2–3 show the average unstandardized coefficient and standard error across the estimated models. Column 4 shows the percentage of coefficients that are estimated to be above zero (for positive mean coefficients) or below zero (for negative mean coefficients), i.e., the cumulative distribution function. Column 5 shows the percentage of statistically significant regression coefficients across the estimated models. In line with Sala-i-Martin (1997), variables are considered robustly associated with SWB when the CDF (0) is at least 95 and the fraction significant at least 90. Regressions with all possible combinations of variables are performed, with the five variables that are exogenous to SWB (age, age squared, gender, immigrant, and year dummies) included as ‘free’ variables in all models. Regressions are conducted with robust standard errors clustered at the regional level. The reported results are based on the generic model (no assumption that the regression coefficients are normally distributed across models) with equally weighted regression models. The results remain very similar when giving more weight to better fitting models or when assuming a normal distribution

Multidimensional measures of well-being and happiness have been used for some time to better track changes in the quality of life in Colombia (Krauss & Graham, 2013) and the government frequently conducts happiness surveys to gauge the state of the nation (Martínez, 2018). Colombia stands out with its high average happiness rank for its level of development (Fig. 1). According to the Gallup World Poll, during the period from 2010 to 2018, Colombia ranked on average 37th out of 156 countries in terms of SWB (6.34/10) but only 73rd out of 156 countries in terms of GDP per capita (PPP) based on the World Development Indicators for the same period. Colombia is not an anomaly in Latin America. Many Latin American countries score higher than expected for their per-capita income levels (Fig. 1). This empirical fact is known in the happiness literature as the ‘Latin American phenomenon’ (Rojas, 2016) and it is explained by Rojas (2016) with the boost to SWB from the quantity and quality of close social relationships and family life in this part of the world (Rojas, 2016, 2018) as well as the generally optimistic nature of Latin Americans (Yamamoto, 2016).

Despite having a relatively high experienced quality of life, Colombia and other Latin American countries have high levels of inequality in SWB (Fig. 2; cf. Helliwell et al., 2016). Measured as the standard deviation of SWB using the Veenhoven and Kalmijn (2005) method, SWB inequality is a broader equivalent to monetary measures of economic inequality as it captures dispersion in subjective valuations of a broader set of factors that influence the quality of life in a country. It tells us how much individuals in a society differ in their self-reported SWB levels. Colombia ranks 20th out of 156 countries in terms of SWB inequality, indicating that SWB levels vary considerably more across surveyed Colombians than across people in European and Anglo-Saxon nations.^3^ The high inequality in SWB is not surprising given the high income inequality in Colombia and the significant associations between income and SWB in Colombia (e.g. Graham & Krauss, 2013; Hurtado, 2016) and in Latin America (e.g., Helliwell et al., 2010; Tay & Diener, 2011; Opfinger, 2016). Studies also find a strong negative association between income inequality at the country level and individual SWB in Latin America (Graham & Felton, 2006; Rojas, 2020).

One distinct aspect of inequality in perceived welfare in Colombia is the high degree of spatial inequality (Fig. 4). SWB in the cities of Bogotá, Caldas, and Quindio are well above those in European nations like France and Spain. At the same time, SWB levels in the peripheral regions of Chocó, Nariño and Putumayo are below those of poorer Latin-American nations like Bolivia and Paraguay. Specifically, there seems to be a large urban-rural divide in Colombia (Burger et al., 2020), where the percentage of thriving people (i.e., those with SWB equal or larger than 7) in predominantly rural areas (39%) is considerably below the percentage of thriving people in urban areas (57%). Part of these SWB differences can be explained by high degrees of economic inequality in Colombia, as highlighted in the most recent World Inequality Report (2018), as well as considerable differences in economic development across Colombian regions (OECD, 2020). The richest Colombian regions have development levels of high-income countries like Chile and Uruguay, while the poorest regions are in this regard similar to lower-middle income countries. However, the suggestion by Rojas (2019) that the relationship between income and SWB is weaker in Latin America than in other parts of the world implies that other factors may play a role in explaining these spatial differences.

The objective of this paper is to empirically test the direction and strength of association of a range of objective and subjective factors with SWB and examine the differences in SWB across individuals and space in Colombia. The investigation of these issues makes important contributions. First, by focusing on experienced welfare along the distribution, this research can help differentiate between the life domains that have sizable and significant effects on experienced welfare of different groups and identify policy areas with the greatest potential to reduce well-being inequality. For example, if it turns out that job opportunities are more important for enhancing the SWB of the bottom 20% of Colombians than access to public transportation, it might be advisable to prioritize generating new job opportunities, if the objective is to improve welfare equity in Colombia. This analysis also helps identify the groups of people with lowest perceived welfare and the reasons why these groups experience lower welfare than other groups. The findings of the study could therefore help rethink development strategies and prioritize future policy reforms.

Second, identifying the policy domains that underpin experienced welfare in Colombia is important for understanding and addressing the risks of social unrest. The recent literature shows that declines in SWB have been associated with revolutions (Arampatzi et al., 2018), peaceful protests (Witte et al., 2020) and voting losses for the incumbent party (Bravo, 2016; Liberini et al., 2017; Ward, 2020) and can predict these phenomena better than standard macroeconomic indicators (Witte et al. 2020). The high spatial inequality in well-being is also problematic because in Colombia it overlaps with ethnic inequality, which has been identified as a risk factor for civil conflict (Montalvo & Reynal-Querol, 2005; Esteban et al., 2012). Indigenous people and Afro-descendants have lower SWB than other groups in Colombia^4^ and are predominantly located in peripheral regions,^5^ where SWB levels are considerably below those in core regions (Fig. 3). Colombia has recently been marked by considerable social unrest and problems linked to crime, violence, and displacement. Growth was weak before the COVID-19 pandemic, which has struck the country hard, affecting both the lower and middle classes (see e.g., Espinel et al., 2020; Gonzalez-Diaz et al., 2020; Lustig et al., 2020). Criticisms of underinvestment by the government in health care and education, the slow implementation of the 2016 peace accords, and the Venezuelan migration crisis have grown too, adding to the list of grievances that could have a significant and negative effect on perceived welfare in Colombia and social stability.

Third, this study wishes to take a broad look at SWB in Colombia. Most studies on this topic in Colombia focus on specific dimensions of well-being or the well-being of specific groups. For instance, Velásquez (2016) studies the importance of social relations and participation in social life in the city of Manizales, while Moreno-Sánchez et al. (2018) examine the gains in SWB of a poverty alleviation program. To our knowledge, the only study that provides a broad overview of SWB in Colombia is the work by Krauss and Graham (2013). They find that improving the SWB of Colombians is conditional on minimizing unemployment spells, improving the provision of health care, and enhancing safety. This background paper extends the work by Krauss and Graham (2013) by (1) exploring a more recent period and using a bigger data set with close to 9000 observations and information on many objective and subjective factors influencing SWB; this allows us to incorporate additional life domains (e.g. digital and road access, income sufficiency and financial struggle, health and social support) and minimize omitted variable bias in previous studies on Colombia, (2) using quantile regressions to explain differences in SWB across individuals and space and the domains that explain these differences, (3) assessing how SWB inequality can be reduced by prioritizing the life domains with the largest potential to increase SWB of the individuals at the bottom of the welfare distribution, and (4) accounting for reverse causality using a Lewbel IV estimator (see e.g., Arampatzi et al., 2018; O’Connor & Graham, 2019; O’Connor, 2020).

Our results confirm the results of Krauss and Graham (2013) on the importance of income, economic security, economic optimism, education and health for boosting experienced welfare in Colombia. Unlike Krauss and Graham (2013) but in line with the findings by Rojas (2016), our results suggest that social support and confidence in national institutions boost SWB in Colombia. Our results corroborate the results in Krauss and Graham (2013) that men, the middle-aged, and rural residents have lower experienced welfare, but in addition, we show the importance of income sufficiency, and digital connectivity. Furthermore, our quantile regressions provide very different insights from those in Krauss and Graham. These regressions reveal that standard-of-living improvements, housing affordability and civic engagement matter more to the most fortunate (i.e., those in the top 20% of the well-being distribution), while having education, sufficient income, economic security, digital connectivity, and a job are much more strongly associated with the well-being of those in the bottom 20% of the experienced welfare distribution. The policy areas that matter more for the least fortunate respondents also explain the majority of spatial differences in perceived welfare between urban and rural areas and between core and peripheral regions in Colombia.

The remainder of this paper is organized as follows. The key concepts and context are discussed in Section 2. We focus on SWB in relation to public policy and the literature on the known correlates of SWB in Latin America and Colombia in particular. Section 3 presents the data on SWB and the independent variables included in the analysis for Colombia. Section 4 discusses the empirical analysis and findings, while Section 5 offers a summary and concluding remarks along with a discussion of caveats.

## Concepts and Context

What is SWB? How is it measured? How does it relate to public policy? SWB or experienced quality of life is often used interchangeably with “happiness”, defined as the “*degree to which an individual judges the overall quality of his/her own life-as-a-whole favourably*” (Veenhoven, 1984, Chapter 2). This definition of happiness refers to cognitive measures of well-being, which have been the focus of most work on SWB in economics (Clark, 2018) and which can be measured using surveys. In this study, we rely on this definition of SWB and measure it using the responses to the Gallup World Poll’s question “*Please imagine a ladder, with steps numbered from 0 at the bottom to 10 at the top. The top of the ladder represents the best possible life for you and the bottom of the ladder represents the worst possible life for you. On which step of the ladder would you say you personally feel you stand at this time?”*

### Subjective Well-Being as a Measure of Experienced Welfare

Cognitive measures of SWB are increasingly perceived as a meaningful and consistent way of capturing experienced welfare or the quality of life in a country (Senik, 2011). Gross Domestic Product (GDP) (per capita) obtained from national accounts data, income or expenditures from household surveys, and multidimensional poverty measures constructed using objective data remain the most widely used quality-of-life indicators. Yet, it is increasingly recognized that these objective measures mainly focus on the *liveability* of the environment or the *opportunities* for a good quality of life, while SWB predominately reflects *life outcomes* that are closely linked to *experienced* welfare or how people appreciate the quality of their own lives.

This distinction between life chances and life outcomes is pivotal (Veenhoven, 2000). Opportunities and outcomes are related, but not necessarily the same. Chances can fail to be realized, while at the same time people can succeed in life despite poor opportunities. Hence, increased income or expenditures do not necessarily indicate rising SWB levels or experienced welfare and increased welfare need not reflect improved incomes or rising expenditures. Public-health research makes a similar distinction between opportunities and outcomes (Veenhoven, 2000). Health indicators reflecting conditions for a good health such as access to health care and adequate nutrition are considered different and used differently from indicators reflecting health outcomes such as disease and mortality figures. For this reason, a substantial amount of health research assesses the relationship between conditions and outcomes. For example, do public expenditures on mental health care really improve mental health? In the literature on quality of life, means and ends are often less well distinguished than in the health-related research and policy discussions equate or proxy life satisfaction with liveability of the environment.

Although subjective data come with their own problems related to the validity and reliability of the measures (see e.g., Bertrand & Mullainathan, 2001),^6^ they can also provide useful information that cannot be obtained from objective data sources (e.g., Veenhoven, 2002; Deaton, 2008; Diener et al., 2009; Ravallion, 2012). First, objective indicators do not capture people’s values and preferences on the extent to which objective conditions in a country matter and translate into outcomes of a good life (Veenhoven, 2000, 2002). Although these domains may contribute to SWB, they do not necessarily do so. Ultimately, the choice to use objective measures as proxies for welfare reflects the subjective opinions of analysts and policy makers of what constitutes a good life (Deaton, 2008). In this case, the problem is not only that it can be presumed that an aggregate monetary indicator or a list of objective conditions can be indicative of quality-of-life outcomes (Dolan & White, 2007), but also that one can decide how to weigh the different aspects represented by objective data when aggregating them into an index (Diener et al., 2009).

Second, SWB data can be used to uncover the objective and subjective conditions that underpin well-being using regression analyses. It has been shown that objective factors such as age, marital and education status, financial situation, and health determine to a large extent an individual’s life satisfaction (Clark, 2019; Stutzer & Frey, 2006; Clark et al., 2008; Graham, 2008; Winkelmann, 2014; Layard, 2011), but subjective factors associated with perceptions and expectations about family relationships, work, community and friends, personal freedom, institutional quality, and personal values are also imperative to individual happiness (Helliwell, 2006). Admittedly, undertaking these regression analyses is not without challenges. Systematic differences have been found in how the different aspects of objective well-being are valued across countries as well as across groups or regions within countries (Diener & Suh, 2000; Deaton, 2008; Diener et al., 2010). Values and preferences within a given society may also change over time as people recalibrate their SWB based on the ‘ideal’ they have for their personal life (‘reference point’). Most notably, the spread of social media and the internet have accelerated these processes in many developing countries. In this regard, Graham (2005: 49–50) has argued that one effect of technological advances and globalization is the “*increasing flow of information about the living standards of others, both within and beyond country borders, which can result in changing reference norms and increased frustration with relative income differences*”.

Third, subjective data include information that is often absent in objective data, which makes them useful as standalone indicators (Veenhoven, 2002; Diener et al., 2009; Jahedi & Méndez, 2014). While objective measures capture the objectively measurable part of a concept, they often fail to include all its relevant components, especially those that refer to difficult-to-quantify issues. Objective indicators are preferred, when clearly defined concepts are being measured. However, with regard to multifaceted and less-easily-defined concepts, such as environmental issues, climate change, governance, social cohesion, and other quality dimensions of development, subjective measures are useful as they factor in the effects of these issues on experienced welfare by gauging people’s evaluations, experiences and expectations (Diener et al., 2009; Jahedi & Méndez, 2014).

In sum, relying only on objective statistics to draw conclusions about experienced welfare can obscure important social and economic developments in countries. In the years prior to the Arab Spring most objective economic indicators showed improvements in development (Arampatzi et al., 2018). They also missed the rise in grievances related to the quality of public services, corruption, and inclusion.

### Subjective Well-Being and Public Policy

Recent studies attempt to inform public policy by examining (1) whether economic and social policy contributes to SWB (evaluating policy), and (2) the costs or benefits in terms of SWB of certain social problems (priority setting in policy). Dillenseger et al. (2019) find that parental leave programs contributed significantly to the SWB of Dutch parents, while Odermatt and Stutzer (2015) establish that smoking bans in Europe hardly affect SWB except for the SWB of smokers who want to quit smoking. Studying the SWB effects of a poverty alleviation program in Colombia, Moreno-Sanchez et al. (2018) conclude that SWB increases for the participants, especially those who entered the program with lower SWB levels. At the same time, Carresco et al. (2020) and Martínez and Maia (2018) only find limited effects of poverty alleviation programs in Uruguay and Colombia, respectively. More generally, research has shown that the quality of institutions and policymaking is positively associated with SWB (Bjørnskov et al., 2010; Rode, 2013; Arampatzi et al., 2019).

SWB data can also be used to prioritize policy areas by examining which types of investment would yield the highest increase in SWB (Frijters et al., 2020). Di Tella, MacCullogh and Oswald (2001) compare the costs of unemployment and inflation in terms of SWB and conclude that unemployment is more damaging to happiness than inflation. Mutual trust and social support from high-quality social networks are strongly related to long-term SWB (Helliwell, 2006; Rodriguez-Pose & Von Berlepsch, 2014), but are undervalued by policy makers (Frijters et al., 2020). A big gain in SWB can also be achieved by boosting investments in mental health care (Layard et al., 2013) and taking measures to tackle unhealthy eating and living patterns (Veenhoven, 2019).

### International Evidence on Correlates of Subjective Well-Being

The main factors underpinning SWB are well known (Diener & Seligman, 2004; Frey, 2010; Layard, 2011; Clark, 2018). Genetic factors and personality traits explain to a large extent SWB (Bartels, 2015; Lykken & Tellegen, 1996). Other factors that play a role include demographic and socio-economic individual characteristics, including age (Clark, 2019), health (Graham, 2008), income (Clark et al., 2008), marital status (Stutzer & Frey, 2006), social contacts (Helliwell, 2006), and employment status (Winkelmann, 2014). In addition, country characteristics such as economic freedom, political freedom, quality of institutions, and tolerance of minorities also matter for quality of life in a country (Veenhoven, 2012). A recent study by Clark (2018) shows in this regard that differences in SWB between countries can be mostly explained by income (37%), health (15%), freedom (11%), and social support (8%). Likewise, Diego-Rosell et al. (2018) find that material and occupational domains explain most of the cross-country variance in SWB around the world.

Many papers show that the correlates of SWB are quite similar across cultures (Helliwell et al., 2009; Tov & Diener, 2013; Diego-Rosell et al., 2018), underscoring the importance of satisfying basic needs such as safety, shelter, good health, and intimate relationships (Veenhoven, 2010). At the same time, there are cross-country variations in the relative importance of different domains. Economic factors are more important for the SWB in poorer countries (Oishi, 2010) and countries with a more materialistic value orientation (Delhey, 2010). Freedom is more strongly associated with the SWB in wealthy and more individualistic countries than in poorer and more collectivistic countries (Oishi et al., 1999; Inglehart et al., 2008). Also in more individualistic cultures personal achievement and self-esteem (Diener et al., 1995) have a stronger association with SWB, while in more collectivistic cultures social harmony (Uchida & Kitayama, 2009) and pursuing goals to make family and friends happy (Oishi, 2010) matter more for happiness.

Diego-Rosell et al. (2018) conclude that in Latin America and the Caribbean material well-being is the most important driver of SWB, although the association between material well-being and SWB is weaker in Latin America than in other parts of the world. Related to this, Rojas (2019) argues that SWB in Latin America is more strongly associated with perceptions about relative income, formed based on comparisons of one’s income with that of the relevant age- and gender-based reference group, than with their actual household income.^7^ Along these lines, Diego-Rosell et al. (2018) argue that satisfaction with income has been overlooked in examining the relationship between income and SWB. Community context is another important domain contributing to SWB in Latin America. This is in line with the findings of Rojas (2016, 2018), who shows that the rich social life in the region explains the relatively high SWB levels in this part of the world.

Likewise, differences can be found within countries, where in the context of SWB inequality the correlations between SWB and different domains vary across the happiness distribution. Lamu and Olsen (2016), Graham & Nikolova (2015) and D’Ambrosio et al. (2020) find that the association between income and SWB is strongest for the unhappiest respondents. This suggests that income redistribution would not only reduce income inequality, but also SWB inequality. The other domains that have the strongest association with the SWB of the unhappiest people in the distribution are health (Lamu & Olsen, 2016) and unemployment (Binder & Coad, 2014). These findings suggest that improvement in basic needs will tend to benefit most the unhappiest members of society.

### Drivers of SWB in Colombia

What do we know about the drivers of SWB in Colombia? Krauss and Graham (2013) examine SWB and differences in SWB in Colombia using data on Cantril Ladder scores for 2010 and 2011 from the Latin America Public Opinion Project (LAPOP). They find that economic factors, including income, unemployment, and education, and economic policy instruments such as pension or health plans – which help to counteract economic insecurity – have a strong and significant association with SWB. In this regard, they find evidence of the importance of both people’s current and future economic situation for shaping SWB levels. Men, middle-aged people, those with children and those in peripheral territories reported significantly lower levels of happiness, while people in good health and those living in safe and healthy environments, with access to water and sanitation, reported higher SWB levels. Not all factors in their model correlated with SWB. Marital status, migrant status, religion, institutional trust and civic engagement were not associated or were associated only in a limited way with Colombians’ SWB levels.

In terms of differences across individuals and areas, Krauss and Graham (2013) find that income, religion and family relations (safety nets) matter more for the SWB of the poor, while discrimination affects more the SWB of the affluent. Looking at differences in SWB correlates in urban and rural areas, the authors find that unemployment and poor public health services had a stronger influence on the SWB of people in rural areas than those in urban areas. Based on their findings, Krauss and Graham (2013) conclude that the policies that would improve SWB in Colombia and at the same time reduce SWB inequality include minimizing the rate and duration of unemployment spells, improving safety, the delivery of public health services and the completion of elementary school programs, ensuring minimum level of household income to make ends meet, and increasing the share of the population with pension and health plans.

The findings by Krauss and Graham (2013) are corroborated by other studies that have focused on SWB in Colombia. Hurtado (2016) finds that income, education and unemployment are important drivers of SWB levels in Colombia, while Hurtado et al. (2017) obtain that informal workers not covered by social security report lower SWB levels than formally employed workers. Lodoño Vélez (2011) shows that Colombians are very optimistic regarding future mobility and finds a positive association between perceptions of fairness in socioeconomic outcomes and SWB. At the same time, very few Colombians (15%) perceive the income distribution as fair. This indicates that income inequality affects SWB not only through economic deprivation of those at the lower end of the distribution, but also through perceptions of feeling left behind. More generally, the importance of subjective economic factors highlights the fact that it is not only absolute but also relative income that matters. These findings indicate that income affects a person’s SWB through three channels associated with a need for sufficiency, status, and fairness. In other words, income is important because it allows a person to make ends meet, to achieve a certain status in society, and to determine whether they are treated fairly in the labor market. Likewise, education and employment are also both related to socio-economic status, from which people derive meaning in life and as well as income and economic opportunities.

Social capital and safety are two other topics that receive attention in the happiness literature on Colombia. Velásquez (2016) analyzes how social relations are associated with SWB in Manizales, while Martínez et al. (2019) find in research on mental health in Cali that general trust in people protects against psychological distress. A recent study by Chica-Olmo et al. (2020) in the city of Medellin finds that respondents who did not feel safe or had to move from their former municipality of residence for extortion, kidnapping, pressure from armed groups, or threat of common delinquency reported a lower experienced quality of life. Using block-level data on homicide rates in Medellin, Medina and Tamayo (2012) find a negative effect of perceptions of insecurity on SWB as well as a negative effect of the neighborhood’s homicide rate for the poorest households. Wills-Herrera et al. (2011) examined the SWB of 742 rural producers in five conflict-affected areas in Colombia and found that perceptions of insecurity are significantly and negatively associated with SWB. Building upon the SWB literature in general and the work by Krauss and Graham (2013) in particular, we next explore the factors underpinning SWB in Colombia and those underlying differences across individuals and space.

## Data and Methodology

The Gallup World Poll (GWP) in Colombia includes exactly 1000 randomly selected respondents, (men and women of 15 years and older) surveyed in each year during the period from 2010 to 2018. In total, our sample includes 9000 observations, whose socio-demographic composition is provided in Table 1. The GWP is designed to be representative at the country level. Typically, the survey covers entire countries, including rural areas, except for unsafe or inaccessible regions in a few countries. Although some areas have been unsafe in recent years due to civil conflict, the GWP in Colombia is reasonably spatially representative, although larger and richer regions tend to be slightly overrepresented (see Table 2).

In this paper, we explore differences between core and peripheral regions and urban and rural areas. The core regions are Antioquia/Eje Cafeter, Bogotá, and the Central East; the periphery contains the North Caribbean, South West, and the National Territory. We use the Gallup classification (see also Easterlin et al., 2011; Burger et al., 2020) based on the respondent’s self-reported type of settlement to define urban and rural areas. There are four self-reported types of settlements: (1) a rural area or farm; (2) a small town or village; (3) a large city; (4) a suburb of a large city. Settlements of types (1) and (2) are considered rural areas,^8^ while those of types (3) and (4) are considered urban areas. Almost 55% of respondents in our sample live in an urban area, a percentage that is comparable to the percentage of people in Colombia that live in places with more than 50,000 inhabitants.^9^

SWB is measured using the “Cantril Ladder” or “Self-Anchoring Striving Scale” (Cantril, 1965). The scores are obtained by answering the question: “*Please imagine a ladder, with steps numbered from 0 at the bottom to 10 at the top. The top of the ladder represents the best possible life for you and the bottom of the ladder represents the worst possible life for you. On which step of the ladder would you say you personally feel you stand at this time?*”

Figure 4 displays the distribution of responses to the Cantril ladder question in Colombia, Latin America and the Caribbean, the Western countries (the Anglo-Saxon world and Europe) and the rest of the world (excluding Latin-America and the Caribbean). The happiness distribution of Colombia resembles closely that of other Latin American and Caribbean countries. Although the average SWB is relatively high in Colombia (see Fig. 1), there are considerable differences in SWB across individuals. A large share of the respondents (38%) evaluate the quality of their life with a score of 5 or lower, but a sizable portion (18%) provide a score of 9 or 10. It is remarkable that the percentage of respondents evaluating their quality of life with a score of 10 is higher than the percentage of those responding with a score of 9. The same phenomenon, observed in other Latin American and Caribbean countries (see also Fig. 4), is likely driven by both cultural response style and high income inequality, as noted by Brulé and Veenhoven (2017). In Western countries, SWB is more equally distributed, with a clustering of scores around 7 and 8 and a lower percentage of people who score a 10.

We include as independent variables a wide range of factors that have been shown to influence the level and inequality in SWB. These include objective and subjective *personal characteristics. Objective personal characteristics* of demographic and economic nature include age, gender, marital status, household composition, per capita household income, migration status, health status, education level, employment status, digital connectivity, place of residence, and civic engagement through donation, volunteering or assisting others in need in the previous month. With regard to education level, we distinguish between 3 categories: (i) individuals with complete or some elementary education (up to 8 years of basic education) (reference category), (ii) individuals with secondary education who finished high school and those with up to 3 years of tertiary education (up to 15 years of education); (iii) individuals with tertiary education who have a college degree or have completed 4 years of education after finishing high school. There are five employment categories: individuals who are full-time paid employees (reference category), self-employed, part-time paid employees, unemployed, and out of the workforce. Digital connectivity is another objective personal characteristic, measured as an index with two equally weighted responses to a question on access to a landline telephone or a mobile phone for personal use and a question on access to the internet.

We include several subjective personal characteristics that have an influence on SWB. These are religiousness, financial struggle in the preceding 12 months, income sufficiency enabling comfortable living, satisfaction with own standard of living, economic expectations, and social support from friends and family. To assess the influence of public policy outcomes on SWB we include a number of subjective domains measuring satisfaction with the quality of the environment (air and water), public services (transportation, road infrastructure, health care), personal freedom, social mobility and efforts to fight poverty, as well as perceptions of housing affordability, safety, corruption, and national institutions. We also control for objective regional conditions by including the regional GDP per capita, regional institutional quality, and the inflow of migrants to the region, measured as the change in the share of migrants between 2015 and 2005. A complete description of the variables included in our analyses can be found in Appendix A.

Figure 5 shows the socio-demographic differences in SWB across respondents with different objective characteristics. The largest differences in SWB are observed between the poorest 20% of residents (5.4 on average) and the richest 20% (7.2), those with only elementary education (5.5) and the college educated (7.2), the unemployed (5.8) and the full-time employed (6.7), and the young (<30 years, 6.8) and the elderly (60+, 5.9). Women report slightly higher SWB scores than men, while people with no partner surprisingly report a higher level of SWB. However, the difference in SWB between those who are single and those who are not disappears after we control for age differences as younger people are less likely to have a partner.

We employ a simple life-satisfaction regression model to examine individual differences in SWB in Colombia:

1 $$ {SWB}_{ijt}=\kern0.75em {X}_{ijt}^{\prime}\beta +{Z}_{jt}^{\prime}\theta +{t}_t+{\varepsilon}_{ijt} $$

In this model, *SWB*_*ijt*_, the reported subjective well-being for individual *i* in region *j* in year *t*, depends on ${X}_{ijt}^{\prime }$ – a vector of objective and subjective individual characteristics – including gender, age, marital status, household income, employment status, health status, self-evaluated religiousness, income sufficiency, economic insecurity, social support, economic expectations and perceived local and national conditions by individual *i* in region *j* and year *t*. *SWB*_*ijt*_ also depends on ${Z}_{jt}^{\prime }$– a vector of regional indicators measured at the department level – including regional GDP per capita, the quality of subnational institutions, and the extent to which the region was affected by the inflow of Venezuelan migrants. A vector of year dummies, *t*_*t*_, controls for time-related shocks common to all regions in the country. We estimate model (1) using the weighted least squares (WLS) estimator with robust standard errors clustered at the regional level. Standardized regression coefficients are estimated to compare coefficients and prioritize policy areas. To address any possible endogeneity bias or reverse causality, which may be a problem because SWB and other included subjective domains are likely jointly determined, we follow Arampatzi et al. (2018) and O’Connor (2020) and re-estimate model (1) using the Lewbel IV estimator as a robustness check.

Apart from an examination of the main correlates of SWB in Colombia, we would like to know how the effects of the different factors and SWB vary along the SWB distribution. Quantile regressions (Koenker & Bassett, 1978) help us understand whether specific factors (e.g. income or social relations) are equally relevant for the individuals with the lowest levels of SWB and the highest level of SWB (Binder & Coad, 2011; Graham & Nikolova, 2015). This helps us to get a better understanding of what matters for whom and to identify the areas that matter most for the happiest and unhappiest individuals in Colombia.

Finally, to examine spatial differences in SWB between (A) urban/core and (B) rural/periphery areas in Colombia, we make use of the Blinder-Oaxaca decomposition analysis (Blinder, 1973; Oaxaca, 1974). The Blinder–Oaxaca decomposition divides the differential of the SWB outcome into two parts: the explained differences in SWB scores between urban/core areas and rural/periphery areas and the unexplained part. More specifically:

2 $$ \Delta \mathrm{SWB}=\left[\mathrm{E}\left({\mathrm{X}}_{\mathrm{A}}\right)\hbox{--} \mathrm{E}\left({\mathrm{X}}_{\mathrm{B}}\right)\right]\prime \upbeta \ast \kern0.75em +\left[\mathrm{E}\left({\mathrm{X}}_{\mathrm{A}}\right)\prime \left({\upbeta}_{\mathrm{A}}\hbox{--} \upbeta \ast \right)\kern0.5em -\kern0.5em \mathrm{E}\left({\mathrm{X}}_{\mathrm{B}}\right)\prime \left({\upbeta}_{\mathrm{B}}\hbox{--} \upbeta \ast \right)\right] $$

- Explained (Q) Unexplained (U)

where ΔSWB is the difference in SWB between the two types of geographical areas, A and B, β_A_ and β_B_ are vectors of coefficients estimated using weighted least squares (using sampling weights) with the respondents located respectively in areas A and B, and β* is a non-discriminatory vector of coefficients, estimated with a pooled regression and used to determine the deviation in the relative importance of each domain in the model between the two groups (A and B). The explained part (Q) – or the “endowments effect” – shows how much of the overall differential in the average SWB can be attributed to differences in the level of the explanatory variables (X) between the two types of areas in Colombia. Hence, this “endowment effect” reflects the differences in local factors and demographics between areas A and B. The unexplained part (U) captures omitted variables as well as changes in the relevance of the estimated coefficients for A and B, respectively.

## Empirical Results

This section discusses the results from different specifications of the main life satisfaction regression model (1), the quantile regressions which allow us to identify the correlates associated with SWB differences along the distribution, and the results from the Blinder–Oaxaca decomposition which allow us to identify the factors correlated with differences in SWB across space.

### Correlates of SWB in Colombia

Table 3 presents results from different specifications of model (1). The first model specification (1), shown in column (1), includes only personal characteristics based mainly on objective data. Compared with the bivariate correlations in Fig. 5, the introduction of control variables into model specification (1) alters the relationship between age and SWB into the expected U-shape, which indicates that SWB declines with age but only up to a point in middle age when it starts increasing again. The female SWB advantage becomes statistically significant, while the SWB gap between immigrants and those born in Colombia is sizable (0.67 on the 0–10 scale), but statistically insignificant because of the low number of immigrants in the sample. Household composition (having a partner or children under 15) is uncorrelated with SWB, but religious people report a slightly higher SWB. This effect may be attributed to the fact that religion is a source of hope and social support. Thus, it is not surprising that the association between religiousness and SWB diminishes when controlling for economic optimism, social support, and civic engagement (see Table 3). Health is a significant and robust correlate of SWB. Even after controlling for having experienced pain yesterday and other personal characteristics, people with health problems score more than half a point lower on SWB than those without health problems. However, it is evident that in specification (1) the coefficient on health picks up other related problems because when we control explicitly for financial struggle, the size of the coefficient on health declines. Education is another significant and robust correlate of SWB. In specification (1), those who completed tertiary education have SWB that is more than one point higher than those who did not complete secondary education. Having a higher income is a strong, positive and robust predictor of SWB, while being unemployed and to a lesser extent being self-employed are negatively associated with SWB. The effect of unemployment disappears after controlling for variables related to a person’s subjective economic situation, indicating that unemployment may induce a financial struggle and lower levels of economic optimism.

How people experience their economic situation is a powerful predictor of SWB. Subjective indicators of an individual’s economic situation explain an additional 9.3% of the variance in SWB in model specification (2). All individual economic indicators remain statistically significant, which shows that a person’s objective economic situation, experienced economic circumstances, and economic optimism have unique associations with SWB. Both indicators of social capital – having social support and civic engagement – are positively associated with SWB, with social support being particularly important in model specification (3).

Among community basics, being satisfied with health care services, housing affordability and road connectivity are the most important correlates of SWB. Safety is positively associated with SWB in model specification (4), although its positive association disappears in the full model (7). Satisfaction with public transportation and water and air quality are not associated with SWB,^10^ but having access to communication (digital access) is a positive predictor of SWB.

At the national level, confidence in national institutions (the government, judicial system, military, and honesty of elections) and the possibility of social mobility are associated with higher levels of SWB (model 5). And so is the perception of having freedom to make one’s choices. However, perceptions of corruption and poverty policies are uncorrelated with SWB.^11^

Regional economic development is not significantly related to SWB beyond its effect through a person’s income and employment status (model 6). Similarly, regional institutional quality is not associated with SWB at the individual level.^12^ However, large inflows of Venezuelans in the region are associated with lower SWB. The effect is small but robust.

In specification (1), the rural-urban SWB gap is 0.47 points while the core-periphery gap is 0.30 points on the 0–10 scale. The rural-urban gap is only slightly reduced to 0.39 points in the full model specification (7) and remains statistically significant while the core-periphery gap is almost halved to 0.16 points and becomes statistically insignificant in the full model specification (7). The factors behind these spatial differences are explored further in the next section.

### Sensitivity Analyses

We assessed the robustness of our findings to alternative model specifications and different sets of conditioning covariates through extreme bounds analysis (EBA) (Leamer, 1983; Sala-i-Martin, 1997; see, e.g., Spruk & Kešeljević, 2016 for an application in a SWB study). EBA tests how robustly each determinant is associated with the dependent variable across all possible regression models with a given set of possible explanatory variables. The results, presented in Appendix D, confirm the general picture from Table 3 that domains that are robustly associated with SWB include some basic personal characteristics (age, gender, education level, being self-employed or unemployed, health, and living in rural areas or the periphery), the subjective economic situation, social capital and digital access, and some community basics (roads and highways, healthcare, and housing affordability).

The linear regressions presented in Table 3 may suffer from endogeneity issues because our dependent variable, SWB, and the subjective domain variables included as independent variables are simultaneously determined. A traditional instrumental variables (IV) estimation, unfortunately, is unattainable due to the absence of valid instruments. Accordingly, we resort to the Lewbel IV estimator (Lewbel, 2012), which specifies an IV estimation using heteroskedasticity-based instruments for cross-sectional data. The Lewbel IV estimator uses internally generated instruments comparable to the difference generalized method of moments (GMM) and the system GMM estimators in panel data research (Arellano & Bond, 1991) to isolate the effect of the individual domain satisfactions on overall SWB.

We first attempted to re-estimate specification (7) in Table 3 by simultaneously instrumenting all the subjective variables. However, this led to a situation of weak instruments caused by the fact that there is not enough heteroskedasticity in the error term to meet the conditions for so many externally generated Lewbel IV instruments. Hence, we proceeded by separately instrumenting each *one* of the subjective variables and re-estimating model specification (7) each time. We provide the results of these Lewbel IV regressions in Table 4, which also compares the WLS and Lewbel IV estimates and reports the extent to which variables are valid and reliable by means of the Kleibergen-Paap rk Wald F statistic and the Hansen J test.

Several subjective domains cease to be significant after re-estimation of specification (7) with the Lewbel IV estimator. The effects of satisfaction with standards of living, civic engagement, satisfaction with roads and infrastructure, and satisfaction with health care become statistically insignificant. Of these variables only the magnitude of the coefficient on satisfaction with standards of living coefficient becomes significantly smaller. One explanation for this finding is that the question related to this variable (‘Are you satisfied with your standard of living’) comes very close to the Cantril ladder question on the best-worst possible life (*Please imagine a ladder, with steps numbered from 0 at the bottom to 10 at the top. The top of the ladder represents the best possible life for you and the bottom of the ladder represents the worst possible life for you. On which step of the ladder would you say you personally feel you stand at this time?*”). The variable gauging the strength of optimism about the economic environment is the only subjective variable that re-gains both its importance and significance with Lewbel IV,^13^ lending support to the thesis that expectations about the prospects of the economy play an important role in shaping SWB in Colombia.

### Prioritizing Policy Areas

It is difficult to determine the relative importance of the different policy areas because some of the variables are differently scaled. Hence, we re-estimated specification (7) in Table 3 to obtain standardized regression coefficients and to compare the relative strength of the various drivers of SWB within the model. These standardized coefficients are the coefficients that you would estimate if the SWB variable and all independent variables were all transformed standard scores (z-scores) before conducting the WLS regression. Because these standardized coefficients are all measured in standard deviations, instead of the units of the variables, they are comparable to one another. Applications of these standardized regression coefficients in other SWB studies include Casas et al. (2015) and De Neve et al. (2018).

The results of the standardized regression analysis for the variables that are statistically significant in Table 3 (Column 7) are presented in Table 5, which shows the subjective variables that become insignificant in the Lewbel IV estimations in italics. The results in Table 5 suggest that areas involving the economy and education are relatively more important in explaining SWB differences. Particularly people’s subjective economic experiences (making ends meet and economic optimism) indicate that people care most about the economy and that this policy area is important to prioritize when trying to improve SWB. In line with earlier work by Diego-Rosell et al. (2018) and Rojas (2019), objective income and employment status are less strongly correlated with SWB. However, it can be expected that people’s subjective experiences can be improved through, for example, the creation of productive employment opportunities and improvements in safety nets. In addition, being located in a rural area and digital access are strongly correlated with SWB while health, social support, confidence in institutions only seem to be of secondary importance. Civic engagement and satisfaction with community basics such as infrastructure and health care are also of secondary importance, in addition to being insignificant in the Lewbel IV estimations.

### Explaining Difference in SWB along the Distribution in Colombia

Are there any systematic differences in the determinants of experienced welfares of the most and least fortunate? To answer this question, we utilize a quantile regression analysis based on a specification which includes only the variables that are statistically significant in one of the seven specifications in Table 3. Stated differently, in the quantile regression analysis we exclude the variables that are insignificant in all specifications in Table 3. As a sensitivity analysis, we also estimate quantile regressions based on Models 1–6 in Table 3. The results for the main analysis are presented in Table 6 and Appendix B, while the sensitivity analysis is presented in Appendix C.

The results in Table 6 indicate that *less fortunate individuals, who are in the bottom 20% of the SWB distribution and tend to be poorer* (see Table 7)*, put a stronger weight on basic needs.* In line with previous research, variables related to socio-economic status, including education, unemployment, income and income insufficiency have a strongest association with the SWB of the unhappiest people in society. The sizes of the coefficient estimates for these variables decrease from the 20th quantile to the 80th quantile. We see the same pattern for other basic needs such as having social support and access to digital connectivity as well as for safety and having a partner in models (1) through (6), which do not include all control variables (see Appendix C).

*Where a person lives matters most to the unhappiest in society,* i.e. *geography particularly affects the SWB of the least fortunate people in a rural area or peripheral area*. For this group of people moving to an urban area in the core regions has the potential to improve SWB. This finding may be related to the fact that cities generally offer better access to some community basics that are not included in the model. Geography matters a lot less to the most fortunate who tend to be affluent and are able to experience a good life in peripheral areas of the country.

*Higher-order needs such as civic engagement* (including volunteering and donating), *housing affordability, and satisfaction with road infrastructure* (only in the sensitivity analysis) have a stronger effect on the SWB of the happiest people in society. The differential effect of standard-of-living perceptions suggests that relative income and social comparisons (i.e. concerns over status) are also more important at the top end of the SWB distribution.

*Women and people who are not active in the workforce (*e.g. *students, the retired, nonworking mothers) are significantly happier when they belong to the top of the SWB distribution.* This can be explained by the fact that the more fortunate are also more affluent. They not only have more leisure time, but their basic needs are met through good pensions and/or support by family.

### Explaining the Spatial Differences in SWB in Colombia

The results from the explained part of the Blinder-Oaxaca decompositions are provided in Fig. 6.^14^ They explain more than 50% of the differences in SWB and suggest that spatial differences in SWB are primarily driven by education, economic circumstances and expectations, and access to services, including digital connectivity and health services.

#### The Urban-Rural Gap

The “endowment effect” in the Blinder-Oaxaca decomposition, which is attributed to differences in the level of explanatory variables in rural and urban areas, explains more than half of the urban-rural gap in SWB. The main factors explaining the “endowment effect” are a *lower education level*, a *deprived economic situation* and *lower economic optimism and digital access*. Table 8 shows that the size of the gaps in these areas are significant. For example, only 8% of people in rural areas have completed tertiary education compared to 17% in urban areas. On average per capita incomes in rural areas are also only half of those in urban areas and the percentage of rural respondents who experience financial struggle is much higher (36%) than those who struggle in urban areas (28%). Other statistically significant SWB disadvantages of rural areas include lower health and health care access. By contrast, people in rural areas derive a statistically significant SWB advantage from their higher confidence in national institutions.

#### The Core-Periphery Gap

Similar factors explain the differences in SWB between Colombia’s core and peripheral regions. The “endowment effect” explains over 70% of the core-periphery SWB gap in Colombia, with the main factors underpinning this effect again attributed to a lower education level, a deprived economic situation, lower economic optimism and digital access, and lower health in peripheral areas. By contrast, people living in peripheral areas have greater confidence in national institutions and higher social capital, which again counterbalances a bit the negative SWB effects of worse socio-economic circumstances. Table 8 shows that the core-periphery gaps in these areas are substantial.

## Concluding Remarks and Policy Implications

Colombia stands out not only with its relatively high average SWB, but also with its high level of inequality in experienced welfare. This paper investigates the reasons behind both the high average level of SWB and the high level of SWB inequality. Using Gallup World Poll data for the period covering most of the 2010s, we find that the perceived welfare of the average Colombian is mainly influenced by conditions and expectations related to economic circumstances and education. Digital access also matters, while having social support, good health, and confidence in national institutions also matter but are of secondary importance. These findings are in line with findings of the Latinobarómetro (2018), which shows that economic problems and employment instability are the problems that Colombians care most about.

We find substantial differences in SWB between groups defined by geography, education, and income. Quantile regressions reveal substantial differences in the domains that matter to those at the bottom and the top of the SWB distribution. Standard-of-living improvements, housing affordability and civic engagement matter more to the happiest top 20%, who also tend to be more educated and affluent. Having education, a job, sufficient income, economic security, and digital connectivity are much more strongly associated with the well-being of the bottom 20% in the SWB distribution. The life domains that matter more to the unhappiest respondents also explain the majority of spatial differences in perceived welfare between residents in urban and rural areas as well as between core and peripheral regions.

To improve equity, policy makers should consider improving the access to and the quality of secondary and tertiary education, increasing opportunities for productive employment, reducing economic insecurity, and improving digital access, especially for residents in rural areas and peripheral regions. Addressing rural-urban and core-periphery gaps in experience welfare may require implementing targeted, space-based policies.

Policy actions aimed at closing gaps in these areas have the potential to increase experienced welfare and reduce inequality in Colombia. Reducing inequality is a priority not only on moral and economic grounds, but also because high spatial inequality overlaps with ethnic and racial inequality in SWB, which pose a risk factor for social stability. Furthermore, the importance of implementing redistribution policies is underlined not only by the finding that income and employment status matter for SWB, but also by people’s perceptions of inequality in these objective conditions (Fig. 7). While the percentage of people who perceive the income distribution as unfair or highly unfair has remained above 80% since 2010, according to Latinobarómetro, income inequality, measured with the Gini index, has steadily declined during the same period (Fig. 7). This is in line with the earlier research by Diego-Rosell et al. (2018) and Rojas (2019), who find that relative income and the subjective experience of income are better predictors of SWB in Latin America and the Caribbean than objective living conditions. On the one hand, the relative importance of the subjective economic variables can be explained by considerable differences in the cost of living across Colombian provinces and cities that are not fully captured by income and employment data. For example, Atuesta and Paredes Araya (2012) show that although income levels in Bogotá are generally higher than in the rest of the country, the cost of living there is also highest. On the other hand, it is also well-known that people’s aspirations, expectations, social comparisons, and tolerance for inequality drive people’s SWB levels (Clark et al., 2008; Ferrer-i-Carbonell & Ramos, 2014). Although Colombians have a generally positive outlook on life – 70% of the population thinks that their life in 5 years will be better than their life now – there is low tolerance for inequality in the country. This in turn suggests that perceptions of income inequality may become a hotbed for social unrest in the country – as explained in the tunnel parable by Hirschman and Rothschild (1973).

The findings presented in this study can help national and local governments in Colombia to prioritize future policy reforms. Examining the effects of specific policies and programs on SWB however is beyond the scope of this study. A growing literature evaluates the effectiveness of small-scale interventions aimed at improving SWB in deprived communities (e.g., Lloyd-Sherlock et al., 2012; Martínez & Maia, 2018; Carrasco et al., 2020). There is also an increasing interest in the relationship between public policy and SWB (e.g., Moreno-Sanchez et al., 2018; Morgan & O’Connor, 2020). These studies indicate that the SWB effects of programs and policies targeting vulnerable populations are not necessarily positive. These studies underscore the importance of procedural utility, i.e. how specific programs and policies are designed and implemented (Stutzer, 2019). Martínez and Maia (2018), for example, examine the effect of the Más Familias en Acción program, a conditional cash transfer program in Colombia targeted at vulnerable populations. Although the program increased satisfaction with health and education, it had negative effects on perceptions of poverty, food and income insecurity. Likewise, Chindarkar (2012) examines the effects of a conditional cash transfer program in Peru and concludes that enrollment in the program can cause feelings of frustration and low self-esteem. A recent Uruguayan study (Carrasco et al., 2020) also finds negative effects of a social intermediation program on SWB through its effect on relative wealth perceptions which were updated as a result the improved knowledge on wealth distribution obtained through the program. These studies highlight the importance of addressing the psychological consequences of poverty (Haushofer & Fehr, 2014) in programs and policy making. More generally, it can be argued that policy makers should pay attention to any potential adverse psychological effects of policies and programs that are aimed at helping the vulnerable in society. They should target not only their physical needs but also their basic psychological needs (autonomy, competence and relatedness) (Ryan & Deci, 2000; Stutzer, 2019). How to design such policies and programs should be addressed in future research.

## Footnotes

1 See https://wellbeingeconomy.org/#movement.

2 In this paper we use interchangeably SWB, life satisfaction, happiness and perceived/experienced welfare.

3 Colombia ranks 16th out of 156 countries when we use the alternative method of measuring SWB inequality that was proposed by Delhey and Kohler (2011).

4 While information on ethnicity and race is not available in the Gallup World Poll, it is included in the Latinobarómetro which indicates that the percentage of satisfied Afro-descendants and indigenous people are 10 percentage points lower than the percentage of satisfied white, Asian and Mestizo groups.

5 In Colombia, Afro-descendants are concentrated in the coastal regions, while Amerindians are located primarily in the eastern periphery of the country. According to the 2005 census, indigenous people are located in the departments of Amazonas, La Guajira, Guainía, Vaupes, and Vichada, with secondary concentrations in the departments of Sucre, Córdoba, Chocó, Cauca, Nariño and Putumayo. Chocó is the department with the largest concentrations of Afro-descendants in Colombia, followed by San Andrés, Bolívar, Cauca, Valle del Cauca, with secondary concentrations (10–18%) in Atlántico, Córdoba, Magdalena, Nariño, Antioquia, La Guajira, Cesar and Sucre.

6 In terms of validity, self-reported well-being data have been criticized for reflecting other phenomena that may affect these subjective measures. Specifically, answers to subjective questions would reflect people’s normative notions and desires, instead of the degree to which respondents are satisfied with their life-as-a-whole or with a certain life domain. Therefore, subjective data might suffer from systematic biases from unrelated sources (Bertrand & Mullainathan, 2001). Subjective data have also received some criticism for their reliability. Apart from the fact that the scales on which the subjective variables are typically measured are rather imprecise, responses can be inconsistent and dependent on the mood and reference point of the respondent (Podsakoff et al., 2003). In addition, respondents would have the tendency to conform to social desirability and answers can be affected by psychological factors, such as personality (Bertrand & Mullainathan, 2001; Redelmeier et al., 2003).

7 This finding is also confirmed by the work of Macchia et al. (2019), who found that the rank of income more strongly predicts well-being in more unequal countries.

8 Compared to other studies (e.g. Easterlin et al., 2011; Burger et al., 2020), we classify peri-urban areas as rural areas. Hence, when we speak of rural areas, this encompasses (1) rural areas or farms and (2) a small town or village.

9 At present, most studies that examine urban-rural differences in SWB utilize the respondent’s own assessment of the type of place they live in. Recently, six international organizations (the EU, FAO, ILO, OECD, UN-Habitat and the World Bank) have developed a more objective degree of urbanization measure, which has been applied to the Gallup World Poll by overlaying the interview geotags against this geospatial layer (Dykstra & Papadimitriou, 2020). However, as the interview geotags were only available for the years 2016–2018, we do not use this measure of urbanization in this study. A comparison of urban-rural SWB using the perceived urbanization level and the degree of urbanization measure for Colombia shows only very limited differences in urban-rural SWB by measurement method (Burger et al., 2020).

10 In unreported results we also verified that SWB was unrelated to objective pollution and satisfaction with environmental preservation.

11 We additionally explored perceptions of youth development. SWB was not positively associated with satisfaction with the educational system and schools and the opportunity for children to learn and grow in Colombia.

12 We observed a sizeable bivariate correlation between SWB and institutional quality at the regional level (r = 0.54) but not when analyzed at the individual level (r = −0.03). The main reason is that the two largest discrepancies occurred in two of the three most populous regions (Bogota and Valle del Cauca), which both scored high on SWB but low on institutional quality (3rd versus 21st for Bogota and 8th versus 22nd for Valle del Cauca).

13 This variable was significant and has a coefficient of comparable size in model specification (2) in Table 3.

14 The unexplained part of the Blinder-Oaxaca decomposition is estimated, but not shown as differences between the core and periphery coefficients and urban and rural coefficients are mostly insignificant. For the explained part, the average coefficients over both groups (i.e. core-periphery, urban-rural) are used.
